# Vacancy defect-promoted nanomaterials for efficient phototherapy and phototherapy-based multimodal Synergistic Therapy

**DOI:** 10.3389/fbioe.2022.972837

**Published:** 2022-08-25

**Authors:** Xinyu Xiong, Li Wang, Shan He, Shanyue Guan, Dawei Li, Mingming Zhang, Xiaozhong Qu

**Affiliations:** ^1^ School of Light Industry, Beijing Technology and Business University, Beijing, China; ^2^ Key Laboratory of Photochemical Conversion and Optoelectronic Materials, Technical Institute of Physics and Chemistry, Chinese Academy of Sciences, Beijing, China; ^3^ University of Chinese Academy of Sciences, Beijing, China; ^4^ Senior Orthopeadics Department, The Forth Medical Center, Chinese PLA General Hospital, Beijing, China; ^5^ PLA Strategic Support Force Characteristic Medical Center, Beijing, China

**Keywords:** nanophotosensitizers, microstructure, vacancy defect engineering, phototherapy, multimodal synergistic phototherapy

## Abstract

Phototherapy and multimodal synergistic phototherapy (including synergistic photothermal and photodynamic therapy as well as combined phototherapy and other therapies) are promising to achieve accurate diagnosis and efficient treatment for tumor, providing a novel opportunity to overcome cancer. Notably, various nanomaterials have made significant contributions to phototherapy through both improving therapeutic efficiency and reducing side effects. The most key factor affecting the performance of phototherapeutic nanomaterials is their microstructure which in principle determines their physicochemical properties and the resulting phototherapeutic efficiency. Vacancy defects ubiquitously existing in phototherapeutic nanomaterials have a great influence on their microstructure, and constructing and regulating vacancy defect in phototherapeutic nanomaterials is an essential and effective strategy for modulating their microstructure and improving their phototherapeutic efficacy. Thus, this inspires growing research interest in vacancy engineering strategies and vacancy-engineered nanomaterials for phototherapy. In this review, we summarize the understanding, construction, and application of vacancy defects in phototherapeutic nanomaterials. Starting from the perspective of defect chemistry and engineering, we also review the types, structural features, and properties of vacancy defects in phototherapeutic nanomaterials. Finally, we focus on the representative vacancy defective nanomaterials recently developed through vacancy engineering for phototherapy, and discuss the significant influence and role of vacancy defects on phototherapy and multimodal synergistic phototherapy. Therefore, we sincerely hope that this review can provide a profound understanding and inspiration for the design of advanced phototherapeutic nanomaterials, and significantly promote the development of the efficient therapies against tumor.

## Introduction

Cancer, also known as malignant tumor, is one of the major diseases that seriously endanger human health and life (Fan et al., 2017; Parchur et al., 2018). According to the latest medical statistics of the World Health Organization (WHO), cancer has become the second deadliest disease in the world, killing about 10 million people every year. Therefore, the effective diagnosis and treatment of cancer is not only related to human life and health as well as quality of life, but also related to the sustainable development of economy and society. At present, the diagnosis and treatment of malignant tumors mainly rely on traditional methods such as surgical resection, chemotherapy and radiotherapy (Yang et al., 2014; Fan et al., 2017; Zhao et al., 2019). Although they can inhibit the development of tumors to a certain extent, there are still some urgent problems to be solved, such as poor treatment effect, large side effects, easy recurrence and metastasis, easy tolerance and so on (Fan et al., 2017; Zhao et al., 2019). Therefore, the developing more timely, efficient and precise diagnosis and treatment strategies are very important to boost the therapeutic efficacy on cancer and improve patients’ quality of life.

In recent years, with the rapid development of nanotechnology, the emerging phototherapy as a non-invasive light-excited cancer therapeutics, including photodynamic therapy (PDT), photothermal therapy (PTT) and multimodal synergistic phototherapy, has been continuously explored and studied to improve the cancer therapeutic outcomes (Cheng et al., 2014; Liu Y. et al., 2019; Liu et al., 2021). Preclinical studies have confirmed that phototherapy is very promising to achieve accurate diagnosis, targeted recognition and efficient treatment for tumor, which provides a new opportunity to overcome cancer as soon as possible (Cheng et al., 2014; Liu Y. et al., 2019). PDT involves three essentials, i.e., light, photosensitive nanomaterial (nanophotosensitizer), and oxygen (Chatterjee et al., 2008). Among them, nanophotosensitizers can passively accumulate and preferentially remain at the tumor sites via the enhanced permeability and retention (EPR) effect (Maeda et al., 2013; Fan et al., 2017; Zhao et al., 2019). Under the excitation of certain wavelength light, nanophotosensitizers can be selectively activated to produce cytotoxic reactive oxygen species (ROS) in tumor rather than normal tissue, so as to induce cancer cell death and achieve tumor-specific PDT (Cheng et al., 2014; Lu et al., 2015; Fan et al., 2017). Compared with PDT for ROS-induced tumor cell apoptosis through light activation of nanomaterials, PTT is another phototherapy paradigm which utilizes nanomaterials with photothermal conversion effect to generate cytotoxic heat (>45°C) for thermal ablation of tumour (Liu Y. et al., 2019; Guiju et al., 2021). In addition, multimodal synergistic phototherapy (including synergistic PTT and PDT as well as combined phototherapy and other therapies) based on multifunctional nanomaterials can achieve synergistic enhancement of tumor treatment (Cheng et al., 2014; Fan et al., 2017; Liu Y. et al., 2019; Zhao et al., 2019). Notably, the phototherapy is a nanomaterial structure sensitive process, and the effectiveness of phototherapy depends largely on nanomaterials (Cheng et al., 2014; Fan et al., 2016; Lu L. et al., 2018; Liu Y. et al., 2019; Yang et al., 2019; Liu et al., 2021). The ideal nanomaterials for efficient phototherapy should include four key properties (Cheng et al., 2014; Fan et al., 2016; Fan et al., 2017; Liu Y. et al., 2019; Zhao et al., 2019; Liu et al., 2021): 1) It should have strong optical response in the near-infrared (NIR) region, allowing the deeper tissue-penetration and lower light scattering to effectively activate the optical-sensitive nanoparticles accumulated in tumors. 2) It should possess high photoinduced ROS production efficiency (ROS quantum yield) or/and high photothermal conversion efficiency in NIR region, so as to acquire satisfactory treatment efficacy of phototherapy. 3) It should be multifunctional and can be coupled with other therapies (e.g., chemotherapy, radiotherapy, gas therapy, chemodynamic therapy, and sonodynamic therapy), so as to overcome the disadvantages of monotherapy and significantly enhance the comprehensive efficacy for complex tumors. 4) In addition, it should also show low toxicity but high biocompatibility to realize minimized side effects. Recently, thus, a great deal of efforts have been made to develop novel phototherapeutic nanomaterials with well-defined particle sizes, morphology, and compositions, such as various noble metal, semiconductor, and carbon-based nanomaterials (Gao et al., 2018; Gu et al., 2019; Melo-Diogo et al., 2019; Wang L. et al., 2020; Zhou et al., 2020; Chang et al., 2021; Tian et al., 2021; Wang L. et al., 2021; Zhou C. et al., 2021). So far, however, the rational design and controllable synthesis of phototherapeutic nanomaterials in order to satisfy the above four properties simultaneously remains a vitally important and fundamental scientific problem.

**SCHEME 1:**
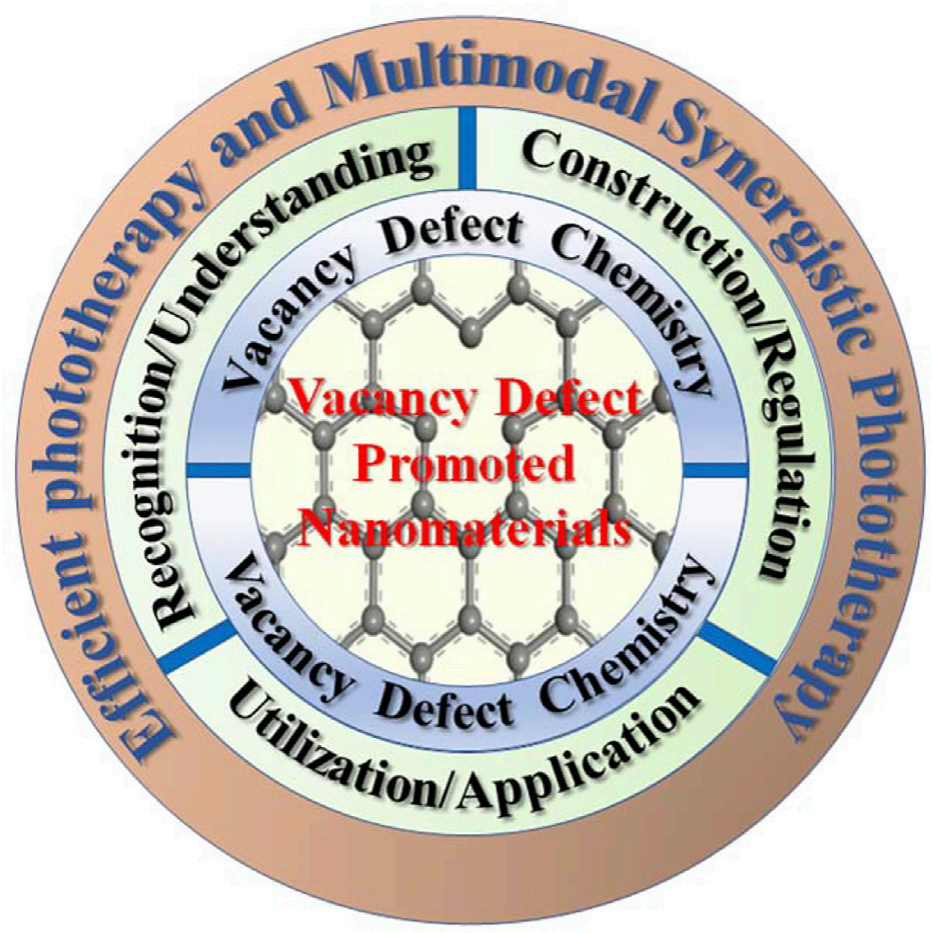
Schematic illustration of vacancy defect-promoted nanomaterials for efficient phototherapy and multimodal synergistic phototherapy.

The properties/performances of phototherapeutic nanomaterials (e.g., optical, photo-dynamic, photo-catalytic, photo-thermal, and biological properties/performances) are in principle determined by their microscopic electronic/band structure and geometric structure (i.e., atomic arrangement) (Coughlan et al., 2017; Huang et al., 2017; Nosaka and Nosaka, 2017; Chen et al., 2021; Liu et al., 2021). Vacancy-type defect of crystals provides the promising opportunity to effectively regulate microstructure and properties in various nanomaterials for phototherapy (Coughlan et al., 2017; Nosaka and Nosaka, 2017; Lu L. et al., 2018). In past decades, it has been recognized that vacancy-type defects, including but not limited to oxygen vacancy, metal vacancy, carbon vacancy, and sulfur vacancy, ubiquitously exist in solid materials, especially in nanostructure materials with small scale (Banhart et al., 2011; He et al., 2013; Paier et al., 2013; Coughlan et al., 2017; Nosaka and Nosaka, 2017; Xu et al., 2017; Lu L. et al., 2018; Yan et al., 2018). In recent years, with the deepening of the research on vacancy-type defects, it can be found that vacancy defects play a significant impact on the physical and chemical properties of phototherapeutic nanomaterials (e.g., optical, photochemical, semiconductor, and plasmonic properties) because they can greatly influence and directly change the microstructure of nanomaterials (Wang X. et al., 2019; Guan et al., 2019; Liu H. et al., 2020; Cai et al., 2021). Meanwhile, vacancy defect-engineering has been explored as a very significant and effective strategy to regulate microstructure and properties of phototherapeutic nanomaterials (Liu H. et al., 2020; Bai et al., 2021; Wen et al., 2021; Zhou B. et al., 2021). Thus, more and more attention have been paid to the influence of vacancy defects on microstructure and properties of phototherapeutic nanomaterials, and constructing and regulating vacancy defects has become an important development direction to achieve efficient nanomaterials and improve their phototherapeutic effect against tumor. Recently, the roles of vacancy defects in phototherapeutic nanomaterials have been widely studied and many vacancy defect-promoted nanomaterials have also been synthetized, showing enhanced phototherapeutic efficacy (Wang X. et al., 2019; Guan et al., 2019; Liu H. et al., 2020; Bai et al., 2021; Cai et al., 2021; Wen et al., 2021; Zhou B. et al., 2021). However, due to the diversity and complexity of vacancy defect structures in nanomaterials (e.g., different concentrations, distributions, and types), these influences of vacancy defects on the microstructure and the phototherapeutic efficacy of phototherapeutic nanomaterials still need to be systematically studied and clearly identified. At the same time, more reasonable design and controllable preparation strategy for vacancy defective nanomaterials need to be further developed to achieve efficient phototherapy. Herein, this review systematically summarizes the recent understanding, construction, and application of vacancy defects in phototherapeutic nanomaterials (as shown in Scheme 1). Starting from the perspective of defect chemistry and defect engineering, we also review the types, structural features, and physicochemical properties of vacancy defects in phototherapeutic nanomaterials. Finally, we focus on the vacancy defective nanomaterials (including oxygen, sulfur, metal and carbon vacancy defective nanomaterials) recently applied in phototherapy, and discuss the significant effects of vacancy defects for phototherapy. This review aims to provide useful vacancy engineering strategies for the design and preparation of advanced phototherapeutic nanomaterials.

## Vacancy defect chemistry and engineering for phototherapeutic nanomaterials

### Vacancy defect chemistry for phototherapeutic nanomaterials

**FIGURE 1:**
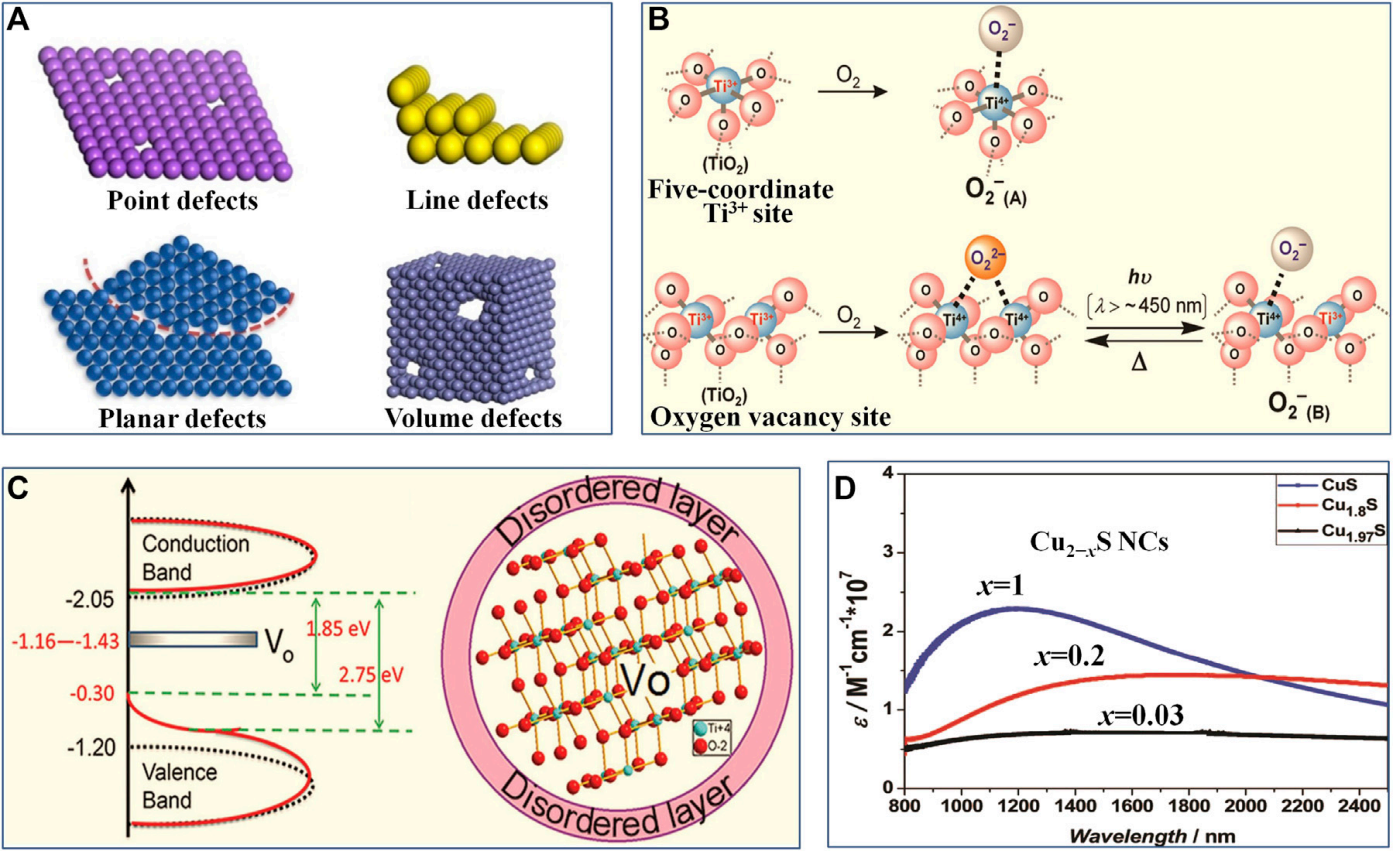
**(A)** Schematic of various defects in crystals. Reproduced with permission from Xie et al. (2020). Copyright © 2020 American Chemical Society. **(B)** Illustrations of O_2_ adsorption on partially reduced TiO_2_ surface to form •O_2_^−^ (adsorbed type A) and O_2_ adsorption on oxygen vacancy defective TiO_2_ surface to form •O_2_^−^ under light irradiation (adsorbed type B). Reproduced with permission from Komaguchi et al. (2010). Copyright © 2010 American Chemical Society. **(C)** Schematic density of states for TiO_2_ P25 Degussa (black dotted line) and black TiO_2_ nanoparticle with oxygen vacancies (red line), Vo represents the localized states created by oxygen vacancies in black TiO_2_ nanoparticle; illustration of the microstructure of black TiO_2_ nanoparticle with oxygen vacancies, Vo represents the oxygen vacancy. Reproduced with permission from Naldoni et al. (2012). Copyright © 2012 American Chemical Society. **(D)** NIR spectra of Cu vacancy defective Cu_2−x_S. Reproduced with permission from Zhao et al. (2009). Copyright © 2009 American Chemical Society.

The phototherapy is a nanomaterial microstructure sensitive process (Cheng et al., 2014; Fan et al., 2016; Lu L. et al., 2018; Liu Y. et al., 2019; Yang et al., 2019; Liu et al., 2021). Generally, defect structures can directly and effectively regulate microstructure of nanomaterials, and they are easily formed in nanomaterials by various physicochemical synthesis and modification methods, due to their high surface energy (Banhart et al., 2011; He et al., 2013; Paier et al., 2013; Li et al., 2017; Xu et al., 2017; Yan et al., 2018; Xie et al., 2020; Zhang et al., 2020). With the development of nanotechnology in recent 10 years, defect chemistry of nanomaterials has become a new research field and an important part of the material chemistry, which mainly involves classification, formation, modulation, characterization, and properties of defects in nanomaterials (Banhart et al., 2011; Li et al., 2017; Xie et al., 2020). In defect chemistry, according to the dimensionality of the crystal lattice, defect structures (as schematically shown in Figure 1A) can be mainly classified as point defects (zero-dimensional defects), line defects (one-dimensional defects), planar defects (two-dimensional defects), and volume defects (three-dimensional defects) (Banhart et al., 2011; Li et al., 2017; Xie et al., 2020). Among them, point defects are the main research content of defect chemistry on nanomaterials, while the vacancy-type defects, such as oxygen vacancy, metal vacancy, sulfur vacancy, and carbon vacancy, are the most commonly observed and representative point defect (Banhart et al., 2011; Paier et al., 2013; Jia et al., 2017; Li et al., 2017; Yan et al., 2018; Xie et al., 2020). In terms of formation mechanism, vacancy defect is a typical intrinsic defect, which can be regarded as an intrinsic component like atoms and ions as well as extend from every direction with small or even atomic scale in nanomaterials (Wang et al., 2011; Paier et al., 2013; Li et al., 2017; Chen et al., 2019; Xie et al., 2020; Zhang et al., 2020). When vacancy defects are formed by empty lattice sites due to thermal vibration or nonstoichiometric compositions (stoichiometric deviation), they are thermodynamically stable and are known as equilibrium vacancies (Matsunaga et al., 2003; Banhart et al., 2011; Paier et al., 2013; Li et al., 2017; Xie et al., 2020; Zhang et al., 2020). In contrast, when undergoing non-thermodynamic equilibrium processes, such as high energy particle irradiation, quenching from high temperature, or cold working, so-called non-equilibrium vacancies usually related to Frenkel pairs can be generated (Kostrubiec et al., 2002; Nagai et al., 2003; Banhart et al., 2011; Li et al., 2017; Yan et al., 2018; Xie et al., 2020; Zhang et al., 2020). From the space configuration of vacancy, the monovacancy caused by the absence of one atom in nanomaterials is the simplest vacancy-type defect, while multiple vacancies (e.g., double vacancies and vacancy clusters) can be generated either by merging multiple monovacancy or removing several adjacent atoms (Banhart et al., 2011; Paier et al., 2013; Li et al., 2017; Xie et al., 2020). The existence of vacancy defects in nanomaterials will disturb the chemical state of the surrounding atoms or ions to some extent and induce lattice distortion/strain (Matsunaga et al., 2003; Banhart et al., 2011; Wang et al., 2011; Paier et al., 2013; Han et al., 2014; Dai et al., 2016; Li et al., 2017; Xie et al., 2020). This, thus, can effectively change the local bond, atomic coordination and electronic properties around specific atomic positions located at vacancy, including bond breaking and re-forming, the bond length, bond energy, electronic compensation, charge and energy density, potential trap depth, and gap states at the Fermi level (Matsunaga et al., 2003; Banhart et al., 2011; Wang et al., 2011; Paier et al., 2013; Dai et al., 2016; Li et al., 2017; Xie et al., 2020). In particular, vacancy defects bring some unpredictable properties, such as outstanding optical, optothermal, optoelectronic, semiconductor, and plasmonic properties (Han et al., 2014; Dai et al., 2016; Li et al., 2017; Ni et al., 2017; Xu et al., 2017; Liu H. et al., 2020; Zhang et al., 2020; Cai et al., 2021). For instance, the formation of oxygen vacancies in semiconductor nanomaterials can induce defect energy levels and thus effectively regulate their electronic structures and band gap states, enhancing substantially their PTT performances (Zhou et al., 2022). Moreover, oxygen vacancy as the active site plays an important role in improving the O_2_ activation and oxygen species’ migration (as shown in Figure 1B), which are crucial for PDT process (Komaguchi et al., 2010; Nosaka and Nosaka, 2017; Guan et al., 2019). Therefore, the understanding of vacancy defect chemistry and properties are crucial to rational design of new phototherapeutic nanomaterials with largely enhanced phototherapeutic effectiveness.

Recently, more and more new phototherapeutic nanomaterials (e.g., metal oxide and chalcogenide nanosemiconductors) and their construction strategies have been systematically developed for phototherapy (Cheng et al., 2014; Fan et al., 2016; Coughlan et al., 2017; Huang et al., 2017; Liu Y. et al., 2019; Liu et al., 2021). However, vacancy defects in specific phototherapeutic nanomaterials will show various existence forms and different properties, owing to that the composition, crystal phases, morphology and microstructures of various nanomaterials are often different and diverse (Coughlan et al., 2017; Huang et al., 2017; Nosaka and Nosaka, 2017; Liu et al., 2021). Therefore, studying the contribution and role of different types of vacancy defects to various phototherapeutic nanomaterials has become an important direction in the field of phototherapy. Transition metal nanocompounds especially transition metal oxide and chalcogenide nanosemiconductors with outstanding optical, optothermal, optoelectronic, semiconductor, and plasmonic properties are the most commonly used phototherapeutic nanomaterials (Fan et al., 2016; Coughlan et al., 2017; Huang et al., 2017; Xie et al., 2020; Liu et al., 2021). Different types of vacancy defects can usually be found in transition metal nanocompounds because of their different chemical composition, bonding modes, and crystalline structure (Komaguchi et al., 2010; Coughlan et al., 2017; Ni et al., 2017; Nosaka and Nosaka, 2017; Li et al., 2018; Liu Y. et al., 2020; Wen et al., 2021; Zhou et al., 2022). According to the different atomic charges in transition metal nanocompounds, vacancy defects can be divided into anionic vacancies and cationic vacancies (Li et al., 2017; Yan et al., 2018; Xie et al., 2020; Zhang et al., 2020). Thereinto, oxygen vacancy in transition metal oxide nanomaterials is the most common anion vacancy, while metal vacancy in transition metal chalcogenide nanomaterials is the most typical cation vacancy (Coughlan et al., 2017; Li et al., 2017; Yan et al., 2018; Xie et al., 2020; Zhang et al., 2020). The oxygen vacancies existed in metal oxide nanomaterials (e.g., MoO_3−*x*_ and WO_3*−x*_ nanocrystals) can induce new energy levels (i.e. defect states) associated with electronic transitions in band structure (as shown in Figure 1C), and the electrons are more easily excited from the defect energy levels under certain wavelength light irradiation, so as to boost charge carrier dynamics (Naldoni et al., 2012; Li et al., 2017; Guan et al., 2019; Zhou et al., 2022). In addition, the conduction electrons related to oxygen vacancies can produce local surface plasmon resonance (LSPR) in the near infrared region, which will enhance the near-infrared response of phototherapeutic nanomaterials (Lounis et al., 2014; Jia et al., 2017; Li et al., 2017; Li et al., 2022). These will result in enhanced photoinduced ROS production efficiency (ROS quantum yield) and high photothermal conversion of phototherapeutic nanomaterials. Compared to oxygen vacancies, metal vacancy in chalcogenide nanomaterials (e.g., Cu vacancy in substoichiometric Cu_2*−x*_X, X = S, Se, Te, 0 ≤ *x* ≤ 1) and related high carrier density can cause the strong LSPR and give rise to a plasmonic resonance band in the NIR region (as shown in Figure 1D) (Zhao et al., 2009; Kriegel et al., 2013; Coughlan et al., 2017; Hu et al., 2019; Lai et al., 2019; Zhao et al., 2020). This induces high NIR light absorbance of metal chalcogenides in a relatively wide wavelength range (from NIR to middle infrared window), thus benefiting the PTT and PDT (Zhao et al., 2009; Kriegel et al., 2013; Coughlan et al., 2017; Hu et al., 2019; Lai et al., 2019; Zhao et al., 2020). In addition to transition metal nanocompounds, carbon-based nanomaterials, such as graphene, carbon quantum dots, and derivatives of metal−organic frameworks (MOFs), are also promising candidates as phototherapeutic nanomaterials for phototherapy (Lu L. et al., 2018; Weng et al., 2020; Chen et al., 2021; Tamtaji et al., 2021; Tian et al., 2021). Meanwhile, carbon vacancy defects in carbon-based nanomaterials can greatly affect their configuration and atomic arrangements and effectively regulate the local π-electron system, so as to tailor the global properties of phototherapeutic nanomaterials and to achieve desired PTT and PDT effects (Li et al., 2017; Liu H. et al., 2020; Weng et al., 2020; Chen et al., 2021; Tamtaji et al., 2021). In addition, except for the type, the distribution and concentration of vacancy in phototherapeutic nanomaterials also contribute a lot to their various properties (Luther et al., 2011; Zhou et al., 2015; Li et al., 2016; Coughlan et al., 2017; Zheng et al., 2021). For example, high concentration of metal vacancy in chalcogenide nanomaterials will lead to a lot of non-radiative recombination sites in the surface trap states, which prevents chalcogenides nanomaterials from exhibiting photoluminescence but promotes non-radiative transitions to release heat (Zhou et al., 2015; Coughlan et al., 2017). To sum up, the unique vacancy-induced features largely dominate the microstructure and properties of various phototherapeutic nanomaterials. Therefore, it is of great significance to study different vacancy defect structures and their respective properties as well as the influence of vacancy defects on phototherapy in more depth and detail.

### Vacancy defect engineering strategies for phototherapeutic nanomaterials

With the establishment of some basic theories of defect chemistry, defect engineering for synthesizing various defective nanomaterials with significantly improved and optimized performance has been widely studied, and effectively used in new phototherapeutic nanomaterials (Jia et al., 2017; Yan et al., 2018; Zhang et al., 2020; Bai et al., 2021; Wen et al., 2021; Zhou B. et al., 2021). It is hoped that defect engineering will not only provide a comprehensive insight into the understanding and utilization of defect chemistry in phototherapeutic nanomaterials, but also offer a novel and effective technique to regulate/optimize the microstructure and characteristics of phototherapeutic nanomaterials. To this end, various strategies of defect engineering have been developed, such as vacancies, heteroatomic doping, crystalline form, size effect, dislocations/steps structure effect (Banhart et al., 2011; Jia et al., 2017; Li et al., 2017; Yan et al., 2018; Xie et al., 2020; Zhang et al., 2020). Among them, vacancy engineering strategy is one of the most researched and effective means to synthesize defective nanomaterials and to adjust/optimize their characteristics (Coughlan et al., 2017; Li et al., 2017; Nosaka and Nosaka, 2017; Yan et al., 2018; Xie et al., 2020). For example, vacancy engineering is a very powerful means to modulate band gap structures (Liang et al., 2015; Li et al., 2017). Abundant carbon vacancies are induced through heat treatment of graphite-like carbon nitride. This will not only enhance the mobility of photogenerated charge carriers, but also will extend light absorption into NIR and produce a low and flat potential band. Therefore, vacancy engineering is of great significance for the design of new phototherapeutic nanomaterials, and offer exciting opportunities for controlling their properties and phototherapeutic effectiveness.

**FIGURE 2:**
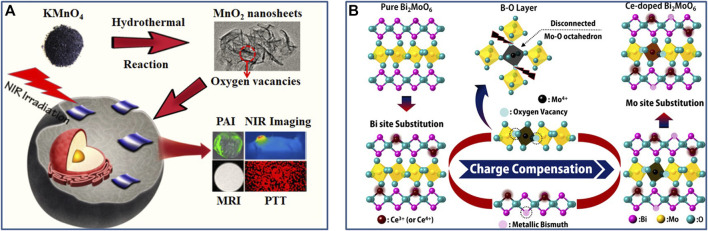
**(A)** Schematic illustration of the preparation of ultrathin MnO_2_ nanosheets with abundant oxygen vacancies through hydrothermal method. Reproduced with permission from Wang L. et al. (2019). Copyright © 2019 American Chemical Society. **(B)** Illustration of the formation process of oxygen vacancies in Ce-doped Aurivillius Bi_2_MoO_6_ structure. Reproduced with permission from Dai et al. (2016). Copyright © 2016 American Chemical Society.

Recent advances in vacancy engineering have enabled researchers to exploit many construction methods and manipulation ways of vacancies with high precision, so as to obtain phototherapeutic nanomaterials with specific microstructure (Komaguchi et al., 2010; Naldoni et al., 2012; Ni et al., 2017; Li et al., 2018; Guan et al., 2019; Hu et al., 2019; Lai et al., 2019; Wang X. et al., 2019; Liu H. et al., 2020; Liu Y. et al., 2020; Weng et al., 2020; Bai et al., 2021; Cai et al., 2021; Tamtaji et al., 2021; Wen et al., 2021; Zheng et al., 2021; Zhou B. et al., 2021; Li et al., 2022; Zhou et al., 2022). The construction methods of vacancy defects in phototherapeutic nanomaterials can generally be divided into two broad categories: 1) directly synthesizing vacancy defective nanomaterials; 2) post treatment of nanomaterials to construct vacancies. In terms of the former method, the specific vacancies can be directly induced with the crystal growth by tuning crystallization conditions and methods, or by regulating the ratio of starting materials, or by elemental doping (Li et al., 2017). For instance, constructing ultrathin two-dimensional nanomaterials with thickness of several atomic layers, by the modulation of synthesis methods and conditions, can induce the formation of abundant vacancies (as shown in Figure 2A) (Wang L. et al., 2019; Peng et al., 2020; Liu et al., 2021; Chang et al., 2022). Guan et al. (Wang L. et al., 2019) found a simplistic hydrothermal method to fabricate an ultrathin MnO_2_ nanosheet with the thickness of ∼1.2 nm, much thinner than that of traditional multilayered MnO_2_ flakes (thickness of ∼60 nm). Importantly, this method can form abundant oxygen vacancies in ultrathin MnO_2_ nanosheets. As obtained ultrathin MnO_2_ nanosheets exhibited highly efficient vacancy-induced PTT effectiveness in the NIR region. Another example, adjusting the stoichiometry or doping (as shown in Figure 2B) is a direct method to create vacancies in multicomponent nanocompounds (Komaguchi et al., 2010; Han et al., 2014; Dai et al., 2016; Ni et al., 2017; Hu et al., 2019; Liu Y. et al., 2020; Zhao et al., 2020; Zheng et al., 2021; Zhou et al., 2022). Representatively, constructing substoichiometric Cu_2*−x*_X (X = S, Se, Te, 0 ≤ *x* ≤ 1) can induce Cu vacancies, and tailoring their stoichiometry can control the concentration and distribution of Cu vacancies (Coughlan et al., 2017; Hu et al., 2019; Zhao et al., 2020; Zheng et al., 2021). This can give rise to enhanced LSPR effect in the NIR region as well as a strong NIR absorption. Moreover, the vacancy defects can be induced by aliovalent doping, and the vacancies density can also be controlled by adjusting dopant concentrations (Feng et al., 2020). Chen et al. (Yu et al., 2017; Yu et al., 2018) designed and fabricated Nb-doped TiO_2_ and Sb-doped SnO_2_ nanocrystals through aliovalent doping, and formed the defect dipoles of dopants and vacancies in these nanocrystals. These nanocrystals showed broad and strong absorption bands in the NIR-II region as well as enhanced photothermal effects, thereby giving rise to excellent photothermal therapeutic efficacy.

The post treatment like annealing, irradiation, ball-milling, etching, and redox is also an efficient and controllable vacancy construction strategy (Banhart et al., 2011; Jia et al., 2017; Li et al., 2017; Yan et al., 2018; Xie et al., 2020; Zhang et al., 2020; Zhou et al., 2022). Heat treatment is one of the simplest and most common post treatment methods. For most metal oxide nanomaterials, annealing will lead to the loss of lattice oxygen, accompanied by the generation of oxygen vacancies (Li et al., 2017; Nosaka and Nosaka, 2017; Xu et al., 2017; Ji et al., 2019; Zhang et al., 2020). Their distribution and concentration can be controlled through changing sintering temperature, heating and cooling rate (Li et al., 2017; Nosaka and Nosaka, 2017; Xu et al., 2017; Ji et al., 2019; Zhang et al., 2020). Another physical method which has been applied for generating vacancies in transition metal nanocompounds and carbon-based nanomaterials is high-energy electrons or ions irradiation (Banhart et al., 2011; Li et al., 2017; Xie et al., 2020; Zhang et al., 2020). Undergoing an electron or ion beam, the atoms can be sputtered away from their lattice sites and thus generate vacancies (Banhart et al., 2011; Li et al., 2017; Xie et al., 2020). Meanwhile, the number and distribution of vacancies can be regulated by controlling irradiation area, energy and time (Banhart et al., 2011; Li et al., 2017; Xie et al., 2020). In addition, this method can also create vacancies with almost atomic selectivity. For example, according to the much lower bonding energy of Sn−O in oxides than Co−O and Fe−O, Wang et al. have selectively created Sn vacancies in CoFeSn oxides through irradiation (Chen et al., 2018). The ball-milling treatment as a facile physical method can also construct vacancies in phototherapeutic nanomaterials (Zhou Z. et al., 2021; Zhou et al., 2022). For example, the mechanical force can transform two dimensional photosensitive materials into thin and short nanosheets during ball milling and create abundant vacancy defects. Etching is a common and efficient chemical post treatment method to construct vacancies (Xie et al., 2020; Zhang et al., 2020). Etching method has mainly utilized the reaction of some chemical components in nanomaterials with added chemicals to produce specific vacancies (Xie et al., 2020; Zhang et al., 2020; Zhou B. et al., 2021). For instance, the phototherapeutic nanomaterials can be etched through alkaline or acid to create metal or/and oxygen vacancies (Sun et al., 2020; Ji X. et al., 2021). Another example, undergoing the oxidative etching with of H_2_O_2_, abundant sulfur vacancies can be induced into MoS_2_ nanosheets (Xie et al., 2020). In addition, the redox process is also a facile chemical post treatment method. Typical example, the annealing of metal oxides is carried out in a reactive atmosphere like H_2_, which will cause hydrogen to react with surface oxygen to produce water and evaporate, thus forming oxygen vacancies (Li et al., 2017; Sun et al., 2019; Zhang et al., 2020; Jiao et al., 2021).

## Vacancy defective nanomaterials-enhanced phototherapy and multimodal synergistic phototherapy

Phototherapy including PDT and PTT is a non-invasive light-induced therapy, which uses NIR-responsive nanomaterials to achieve effective local treatment in tumor sites and could diminish damage to normal tissues (Cheng et al., 2014; Liu Y. et al., 2019; Liu et al., 2021). Moreover, multimodal synergistic phototherapy (including synergistic PTT and PDT as well as combined phototherapy and other therapies) based on multifunctional nanomaterials can achieve synergistic enhancement of tumor treatment (Cheng et al., 2014; Fan et al., 2017; Liu Y. et al., 2019; Zhao et al., 2019). It is worth noting that various nanomaterials have made significant contributions to phototherapy through both improving targeted therapeutic efficiency and reducing side effects (Cheng et al., 2014; Liu Y. et al., 2019; Liu et al., 2021). Nanomaterials for phototherapy are usually composed of inorganic nanoparticles with strong NIR absorption and rapid NIR activation, such as noble metal, chalcogenide, transition metal oxide, polyoxometalate, transition metal carbide, and carbon-based nanoparticles (Wang X. et al., 2019; Liu G. et al., 2019; Gu et al., 2019; Wang L. et al., 2020; Wang L. et al., 2021; Tian et al., 2021). The most key factor affecting the performance of phototherapeutic nanomaterials is their microstructure (i.e., electronic and geometric structures) which in principle determines their physicochemical properties and the resulting phototherapeutic efficacy (Coughlan et al., 2017; Huang et al., 2017; Nosaka and Nosaka, 2017; Chen et al., 2021; Liu et al., 2021). Thus, the microstructure should be rationally designed and precisely tailored to obtain the phototherapeutic nanomaterials with largely improved phototherapy efficacy. With the deepening of the research on defect chemistry and engineering, it is recognized that the vacancy-type defects, such as oxygen vacancy, metal vacancy, carbon vacancy, and sulfur vacancy, commonly exist in phototherapeutic nanomaterials, which can greatly influence and directly change the microstructure of phototherapeutic nanomaterials (Wang X. et al., 2019; Guan et al., 2019; Liu H. et al., 2020; Cai et al., 2021). Constructing vacancy defects in phototherapeutic nanomaterials (i.e., vacancy engineering) provides a very important and effective strategy for modulating their microstructure, controlling their properties, and improving their phototherapy efficacy (Liu H. et al., 2020; Bai et al., 2021; Wen et al., 2021; Zhou B. et al., 2021). Meanwhile, a deeper and more detailed understanding of the influence of vacancy defects on phototherapy is also crucial to rational design of new phototherapeutic nanomaterials. In this section, therefore, we will focus on the representative vacancy defective nanomaterials (including oxygen, sulfur, metal and carbon vacancy defective nanomaterials) recently developed through vacancy engineering for efficient phototherapy (as summarized in Table 1), in order to provide the best possible overview of various vacancy engineering strategies and the effects of various vacancies on phototherapy and multimodal synergistic phototherapy.

**TABLE 1:** Summary of the vacancy defective nanomaterials for phototherapy and multimodal synergistic phototherapy from recent reports in the literature.

Nanomaterials	Vacancy defect type	Methods	Irradiation wavelength (nm)	Irradiation power density (W cm^−2^)	Therapeutic modality	Imaging mode	Cell lines	References
Ultrathin MnO_2_ nanosheets	Oxygen vacancy	Hydrothermal method	808	1.0	PTT	PAI, MAI, and thermal imaging	HeLa cells	Wang L. et al. (2019)
PEG-Na_*x*_GdWO_3_ nanorods	Oxygen vacancy	Chemical mixing procedure at different temperatures and subsequent thermal decomposition	980	1.5	PTT	MAI and thermal imaging	4T1 cells	Ni et al. (2017)
Na_*x*_MnWO_3_-PEG nanorods	Oxygen vacancy	Chemical mixing procedure at different temperatures and subsequent thermal decomposition	980	1.0	PTT	Thermal imaging, PAI, and MRI	4T1 cells	Liu Y. et al. (2020)
]MoO_3−*x*_ nanobelts	Oxygen vacancy	Hydrothermal method, followed by ball milling process and lithium treatment	1,064	0.75	PTT	PAI and thermal imaging	4T1 cells	Zhou Z. et al. (2021)
BiOBr−H/Rub_2_d nanocomposites	Oxygen vacancy	Hydrothermal method and subsequent heat treatment	520	10.0	PDT	FLI	HeLa cells	Guan et al. (2019)
CaAl_2_O_4_:Eu, Nd (CAO) nanosheets	Oxygen vacancy	Combination of high temperature solid state reaction and subsequent wet-grinding and ultrasound treatment	Pre-excitation with 365 nm UV lamp and re-excitation with white LED	--	Self-illuminating PDT	--	4T1 cells	Chang et al. (2022)
B-TiO_2−*x*_ nanoparticles	Oxygen vacancy	Mg-thermic reduction of TiO_2_	808	0.48	Synergistic PTT/PDT	Thermal imaging	B16F10 melanoma cells	Wang X. et al. (2019)
ZrO_2−*x*_@PEG/Ce6 nanoparticles	Oxygen vacancy	Magnesium reduction procedure and subsequent surface modification	660 and 808	0.76 for 660 nm and 2.0 for 808 nm	Synergistic PTT/PDT	FLI and PAI	4T1 cells	Sun et al. (2019)
PEG-MoO_*x*_ nanoparticles	Oxygen vacancy	Hydrothermal method	808 and 1,064	0.75 for 808 nm and 0.6 for 1,064 nm	Synergistic PTT/PDT	Thermal imaging	HeLa cells	Yin et al. (2018)
TiO_2_@red phosphorus nanorods	Oxygen vacancy	Vaporization-deposition method	808	0.85	Synergistic PTT/PDT	Thermal imaging	ccRCC cells	Yang et al. (2021)
CaO-SiO_2_-TiO_2_ (CST) nanocomposites	Oxygen vacancy	Containerless melting technology	808	0.8 W cm^−2^ and 1 W cm^−2^ for NIR irradiation; 10.0 W cm^−2^ for xenon lamp irradiation	Synergistic PTT/PDT	Thermal imaging	LM8 cells	Wang E. et al. (2021)
MnO_2_@Au nanoconstructs	Oxygen vacancy	One-step reduction method	808	1.0	Synergistic PTT/CDT	Thermal imaging	HeLa cells	Peng et al. (2020)
NH−MoO_3−*x*_@BSA nanobelts	Oxygen vacancy	Hydrothermal method, followed by ball milling process, co-intercalation, and surface modification	1,064	0.8	Synergistic PTT/CDT	Thermal imaging	HUVE, HeLa, 4T1 cells	Zhou et al. (2022)
O*x*-POM@Cu nanocomposites	Oxygen vacancy	Chemical mixing procedure at different temperatures	1,064	1.0	Synergistic PTT/CDT	Thermal imaging and PAI	4T1 and HL-7702 cells	Wang et al. (2022)
Black mica (BM) nanosheets	Oxygen vacancy	Combination of calcination, n-butyllithium exchange and intercalation, and liquid exfoliating processes	650 and 808	0.5 for 650 nm and 1.0 for 808 nm	Synergistic PTT/PDT/CDT	FLI, PAI, and thermal imaging	A549, Hela, MCF7, and PC3 cells	Ji et al. (2019)
d-Cu-LDH/ICG nanoparticles	Oxygen vacancy	Coprecipitation method, followed by isomorphic substitution and acid etching	808	0.23 and 0.5	Synergistic PTT/PDT/CDT	FLI	4T1 cells	Sun et al. (2020)
Functional core layers (FLCs) nanosheets	Oxygen vacancy	Wet-chemical exfoliation method based on alkali etching	658 and 808	0.5 for 658 nm and 1.0 for 808 nm	Synergistic PTT/PDT/CDT	FLI, PAI, and thermal imaging	A549, HepG2, CCD-25Lu, HEK 293, and THLE-3 cells	Ji X. et al. (2021)
As/As_*x*_O_*y*_@PDA@M nanosheets	Oxygen vacancy	Coupling ball-grinding with probe sonication-based liquid exfoliating processes and subsequent surface modification	660 and 808	0.3 for 660 nm and 1.0 for 808 nm	Synergistic PTT/PDT/CDT	FLI	A549 and MCF-7 cells	Kong et al. (2021)
WB@hydrogel	Oxygen vacancy	Hydrothermal method and subsequent hydrogenation reaction	1,064	1.5	Synergistic PTT/GT	FLI	4T1 and HepG2 cells	Zhao et al. (2021)
ZrO_2−*x*_@PEG/cRGD (ZPR) nanoparticles	Oxygen vacancy	Modified magnesium (Mg) reduction process	1,064	1.0	Synergistic PTT/SDT	PAI, and thermal imaging	4T1 cells	Jiao et al. (2021)
TiO_2_@TiO_2−*x*_ -PEG nanoparticles	Oxygen vacancy	Aluminum (Al) reduction procedure	1,064	1.5	Synergistic PTT/SDT	Thermal imaging	4T1 cells	Han et al. (2018)
Bi_2_S_3_-Au nanorods	Sulfur vacancy	Solvothermal method and subsequent *in situ* reduction process	808	0.9	PTT	CT and thermal imaging	4T1 cells	Cheng et al. (2018)
FeS_2_ nanoparticles	Sulfur vacancy	Solvothermal method	915	1.0	PTT	Ultrasound imaging, thermal imaging, and MRI	SMMC-7721 cells	Meng et al. (2016)
Bi_2_Se_3_ nanodots	Selenium vacancy	Scalable biomineralization approach	1,064	1.4	PTT	CT, PAI, and thermal imaging	4T1 cells	Wen et al. (2021)
PTh@Au nanoconstructs	Sulfur vacancy	One-step redox strategy	650	50.0	PDT	--	HeLa and U14 cells	Bai et al. (2021)
MoS_2_ quantum dots	Sulfur vacancy	Aqueous room temperature bottom-up synthesis	400–800	10.0	PDT	--	SW480 cells	Ding et al. (2019)
Fe_3_S_4_ tetragonal nanosheets	Sulfur vacancy	Hot-injection thermal decomposition reaction and subsequent surface modification	915	0.5	Synergistic PTT/PDT	Thermal imaging, and MRI	HeLa cells	Guan et al. (2018)
T80-AuPt@CuS nanosheets	Sulfur vacancy	*In situ* reduction process and subsequent surface modification	808	1.0	Synergistic PTT/radiotherapy	CT and PAI	4T1 cells	Cai et al. (2021)
Cu_2−*x*_Se-PEG-SH nanoparticles	Cu vacancy	Aqueous reduction process and subsequent surface modification	808	1.5	PTT	PAI, CT, SPECT, and thermal imaging	4T1 cells	Zhang et al. (2016)
Ultrathin CuS nanocrystals	Cu vacancy	Microwave-assisted synthesis strategy	980	2.48	PTT	FLI	PC-3/Luc+ cells	Zheng et al. (2021)
Cu_3_BiS_3_ nanocrystals	Cu vacancy	Solvothermal method	915	1.2	PTT	CT and thermal imaging	TC71 cells	Li et al. (2015)
Au@Cu_2−*x*_S nanorods	Cu vacancy	Nonepitaxial strategy of tributylphosphine-initialized cation exchange	808 and 1,064	1.0 for 808 nm and 0.7 for 1,064 nm	PTT	--	HeLa cells	Ji et al. (2016)
Ultrasmall Cu_2−*x*_S-PEG nanodots	Cu vacancy	One-step redox strategy and subsequent surface modification	1,064	1.5	Synergistic PTT/CDT	PAI and thermal imaging	4T1 cells	Hu et al. (2019)
CoCuFeSe-PVP-L-Arg nanosheets	Cu vacancy	*In situ* selenylation treatment of precursor	808	1.0	Synergistic PTT/GT	PAI, thermal imaging, and FLI	HepG2 and 4T1 cells	Wu et al. (2021)
Pt–CuS–PNTs nanocomposite	Cu vacancy	Co-assembly, followed by biomineralization process and covalent graft	808	0.5	Synergistic PTT/PDT/chemotherapy	Thermal imaging	B16-F10 cells	Lai et al. (2019)
MoSe_2_/Bi_2_Se_3_@PEG-Dox nanocomposites	Mo vacancy	Ultrasound-assisted exfoliated method and subsequent cation exchange method	808	0.2, 0.5, and 1.0	Synergistic PTT/PDT/chemotherapy	Thermal imaging, PAI, and CT	L02 and HepG2 cells	Wang Y. et al. (2019)
CoFe-mixed metal oxide (CoFe-*x*) nanosheets	Co vacancy	Heat treatment of LDHs precursor	808	1.0	PTT	PAI and MRI	HeLa cells	Wang J. et al. (2020)
W_1.33_C-BSA nanosheets	W vacancy	Solid state sintering, followed by acid etching, exfoliation treatment, and surface modification	808 and 1,064	1.25	PTT	PAI, FLI, CT, and thermal imaging	4T1 and L929 cells	Zhou B. et al. (2021)
5-FU/Cu-LDH nanoparticles	Mg and OH vacancy	Hydrothermal Method, followed by intercalation treatment and acid etching	808	1.0	PTT	MRI and thermal imaging	HCT-116 cells	Li et al. (2018)
Ag@Ag_2_O/LDHs-U nanocomposites	Ag and oxygen vacancy	Modified solvent-free bottom-up approach, followed by *in situ* spontaneous deposition and surface modification	1,064	1.0	PTT	FLI and PAI	L02, HeLa, and HepG2 cells	Li et al. (2022)
Cu@CPP-t nanoparticles	Carbon vacancy	Pyrolysis of Cu-BTC precursor	808	1.6	PTT	PAI and thermal imaging	Hela cells	Weng et al. (2020)
Graphene quantum dots	Carbon vacancy	Magnetic field-assisted solvothermal method	1,064	1.0	PTT	Thermal imaging	4T1, Hela, and H196 cells	Liu H. et al. (2020)
B_4_C@C nanosheets	Carbon vacancy	Hydrothermal method	1,064	1.0	PTT	PAI and FLI	HeLa and HepG2 cells	Guo et al. (2021)
Pd nanozyme	Carbon vacancy	Pyrolysis of Pd@ZIF-8 precursor	1,064	0.28	Synergistic PTT/CDT	--	L929 and 4T1 cells	Chang et al. (2021)
C/Mo_2_C@MoO_*x*_ nanoparticles	Carbon vacancy	Pyrolysis of Mo/ZIF-8 precursor	1,064	1.0	Synergistic PTT/CDT	PAI and FLI	L02 and HeLa cells	Wang L. et al. (2021)

### Anion vacancy-promoted nanomaterials for phototherapy and multimodal synergistic phototherapy

**FIGURE 3:**
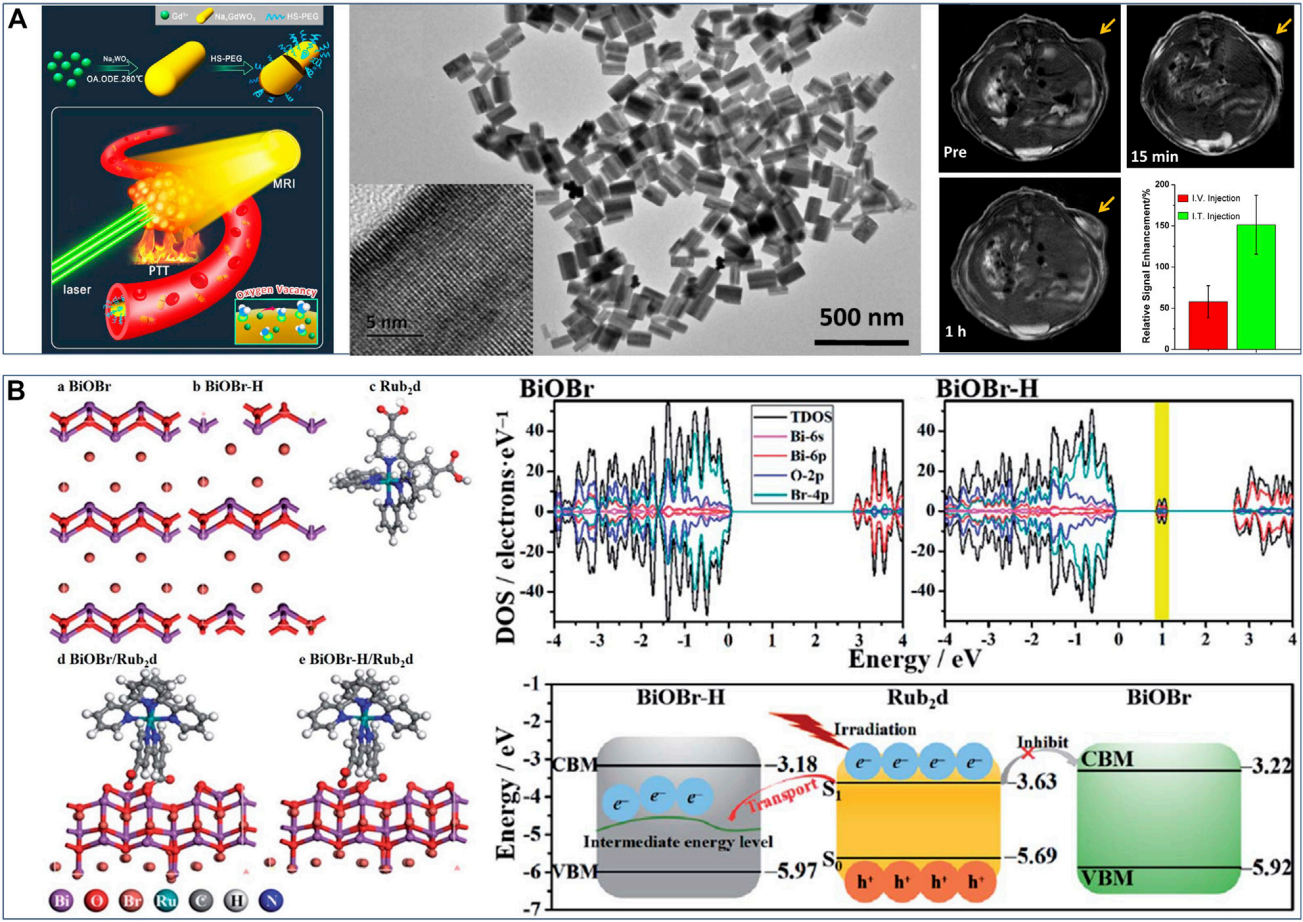
**(A)** Schematic diagram of Na_x_GdWO_3_ nanorods for MRI-guided photothermal, TEM images of Na_x_GdWO_3_ nanorods, and *in vivo* *T*_1_-weighted MR images of 4T1-tumor-bearing mice before and after (I)T. injection of Na_x_GdWO_3_ nanorods. Reproduced with permission from Ni et al. (2017). Copyright © 2017 American Chemical Society. **(B)** Schematic illustration of geometric structure of various BiOBr-based samples and Rub_2_d sample, band structures of BiOBr and BiOBr–H, and the band edge positions of BiOBr, BiOBr–H and Rub_2_d. Reproduced with permission from Guan et al. (2019). Copyright © 2019 The Royal Society of Chemistry.

As a new type of nanomaterials for phototherapy, NIR-responsive metal oxide semiconductor nanomaterials have made great progress in recent years, and show wide application prospect in tumor treatment because of their unique properties (Cheng et al., 2014; Huang et al., 2017; Nosaka and Nosaka, 2017; Liu Y. et al., 2019). Oxygen vacancy as the most common anionic vacancy ubiquitously exists in oxide semiconductor nanomaterials, which is a new state will be formed within the band gap of the O-defected oxide, in which the electrons associated with the metal−O bonds tend to be delocalized, proving to be able to work as the trapping site for photogenerated electron and tends to introduce the occurrence of intermediate energy level (Li et al., 2017; Nosaka and Nosaka, 2017; Wang L. et al., 2019; Guan et al., 2019; Zhou et al., 2022). This can promote the separation of electron and hole, induce band gap narrowing, and produce the related LSPR in the NIR region, greatly affecting the microstructure, properties and PTT efficacy of oxide semiconductor nanomaterials. For example, Guan et al. (Wang L. et al., 2019) reported that the ultrathin MnO_2_ nanosheets with abundant oxygen vacancies were obtained via a simplistic hydrothermal process (Figure 2A). The resulting ultrathin MnO_2_ nanosheets showed significantly enhanced photothermal conversion efficiency (∼62.4%) under 808 nm laser exposure and outstanding photothermal stability. Such a high photothermal performance was attributed to the oxygen vacancies in MnO_2_ nanosheets. The EXAFS data and DFT calculations revealed that the direct band gap semiconductor MnO_2_ can be changed into indirect band gap semiconductor with intermediate energy levels, due to the existence of abundant oxygen vacancies in MnO_2_ nanosheets. The contractible band gap endowed the superb photothermal conversion efficiency of the MnO_2_ nanosheets and great potential in NIR-triggered hyperthermia. Moreover, both *in vitro* and *in vivo* results confirmed the good biocompatible properties and highly efficient vacancy-induced photothermal therapy of MnO_2_ nanosheets. Another example, Bu et al. (Ni et al., 2017) synthesized the oxygen-vacancy defective Gd^3+^-doped Na_*x*_WO_3_ (Na_*x*_GdWO_3_) nanorods by using a facile thermal decomposition approach, with both high-performance MR imaging and outstanding PTT efficacy (Figure 3A). Specifically, oxygen-vacancy-induced small polarons in Na_*x*_GdWO_3_ led to the strong NIR absorption and effective photothermal conversion. Meanwhile, oxygen vacancies could also cause significantly enhanced proton relaxation rates of the Na_*x*_GdWO_3_ (*r*_1_ value of 32.1 mM^−1^ s^−1^ on a clinical 3.0 T scanner). In addition, both *in vitro* and *in vivo* results showed that Na_*x*_GdWO_3_ can be used as an efficient theranostic nanoplatform for MRI-guided photothermal therapy under 980 nm laser irradiation by simply regulating the oxygen vacancy. Thus, the oxygen vacancies were deemed to be responsible for the enhanced MR imaging and efficient PTT treatment. This oxygen vacancy-promoted MRI-guided PTT strategy has certain universality and has recently been extended to Mn^2+^-doped Na_*x*_WO_3_ (Na_*x*_MnWO_3_) system, giving stable MRI and significant PTT performance (Liu Y. et al., 2020).

Compared to the first near-infrared (NIR-I) window (from 750 to 1,000 nm), the second near-infrared (NIR-II) window (from 1,000 to 1,350 nm) for photothermal therapy exhibits inherent advantages of the deeper tissue-penetration, lower light scattering and higher maximum permissible exposure (Lin et al., 2017; Liu Y. et al., 2019; Liu G. et al., 2019; Tian et al., 2021). Thus, many attempts have been contributed to developing new NIR-II-triggered photothermal nanoagents with satisfactory PTT efficacy through oxygen vacancy engineering (Han et al., 2018; Yin et al., 2018; Zhao et al., 2021; Zhou Z. et al., 2021). For example, Chen et al. (Han et al., 2018) created an oxygen-defective TiO_2−*x*_ layer onto the surface of TiO_2_ nanosemiconductors through a facile aluminum-reduction strategy, thus constructing a crystalline-disordered core-shell structure (TiO_2_@TiO_2−*x*_) with black color. Notably, the presence of abundant oxygen vacancies in TiO_2−*x*_ shell endowed these black nanosemiconductors with a high light absorption in the NIR-II window, inducing an enhanced NIR-II-driven photothermal effect and increased photothermal conversion efficacy. Both *in vitro* and *in vivo* evaluations have demonstrated that the photothermal ablation for tumor was enhanced by the black oxygen-defective TiO_2−*x*_ shell, which could completely eradicate the tumors with high biosafety and no obvious reoccurrence. Another example, Tan et al. (Zhou Z. et al., 2021) reported that the dark blue-colored short, thin and defective MoO_3−*x*_ nanobelts were synthetized through a facile ball milling process, followed by a simple lithium treatment, showing efficient PTT performance in the NIR-II window. A combination study including XPS, EXAFS, HAADF-STEM and EPR indicated the generation of abundant oxygen vacancies in layered MoO_3−*x*_ nanobelts by Li intercalation, leading to partial reduction of Mo atoms from Mo^6+^ to Mo^5+^ and thus yielding dark blue-colored MoO_3−*x*_ nanobelts. Such structural changes gave rise to a strong absorption in the NIR-II region and enabled a large extinction coefficient of 18.2 Lg^−1^ cm^−1^ at 1,064 nm, thus causing a high photothermal conversion efficiency of 46.9%. As-obtained MoO_3−*x*_ nanobelts can be used as a nanoagent for photoacoustic imaging-guided PTT to achieve efficient ablation and eradication for tumor under 1,064 nm laser irradiation. Therefore, the oxygen vacancy engineering is a powerful strategy to tune microstructure and properties of phototherapeutic nanomaterials and thus boost their PTT effectiveness for tumor.

Oxygen vacancy engineering strategy can also play an important role in photodynamic therapy. It can effectively regulate the energy band structure of phototherapeutic nanomaterials and significantly improve photoinduced ROS production efficiency. Guan et al. (Guan et al., 2019) reported an oxygen vacancy-promoted BiOBr−H/Rub_2_d nanocomposite for PDT via a two-step procedure: preparation of oxygen vacancy-rich BiOBr (BiOBr−H) by a hydrothermal method and subsequent heat treatment, followed by the coupling of Ru(bpy)_2_C-pyCl_2_ photosensitizer (Rub_2_d). An experimental-computational combination study demonstrated that the strong electronic interactions between BiOBr−H and Rub_2_d resulted in the photogenerated electrons transfer from Rub_2_d to the oxygen vacancy-induced intermediate energy level in BiOBr−H (Figure 3B). Thereby this facilitated rapid electron−hole separation and led to enhanced generation of ^1^O_2_. Specifically, the ^1^O_2_ yield of BiOBr−H/Rub_2_d (0.49) was more than twice that of Rub_2_d (0.22). Furthermore, both *in vitro* and *in vivo* studies confirmed that the BiOBr−H/Rub_2_d nanocomposite was a potent PDT agent for cancer treatment. This strategy of oxygen vacancy-enhanced generation of singlet oxygen also has universal applicability to other photosensitizers (e.g., indocyanine green and zinc phthalocyanine), thus giving great potential in PDT for cancer treatment. In another example of oxygen vacancy-promoted PDT, Feng et al. (Chang et al., 2022) reported that a distinct 2D CaAl_2_O_4_:Eu, Nd persistent luminescence nanosheets (CAO PLNSs) for irradiation-free PDT. A combination study verified the existence of abundant oxygen vacancies in CAO PLNSs, arising from the introduction of Eu^2+^ and Nd^3+^ ions. Notably, the generation of oxygen vacancies in CAO PLNSs induced formation of trap level (i.e., defect energy levels), causing a steady stream of 5*d*-4*f* electronic transition between Nd^3+^ 4*f* and Eu^2+^ 5*d* levels and persistent blue luminescence. Thus, CAO PLNSs can continuously produce cytotoxic ^1^O_2_ through long-lasting self-illuminating PDT without the need for external light excitation, inducing cancer-cell apoptosis. Accordingly, through the systematic evaluation of *in vitro* and *in vivo* experiments, persistent luminescence-enhanced PDT showed outstanding antitumor efficacy. Therefore, oxygen vacancy engineering can not only regulate the energy band structure of phototherapeutic nanomaterials and improve ^1^O_2_ yield, but also enhance the self-luminescence characteristics of phototherapeutic nanomaterials and help the establishment of an efficient self-illuminating PDT systems, so as to overcome the tissue penetration limitation of conventional PDT and provide new avenues for efficient treatment of deep-seated tumors.

**FIGURE 4:**
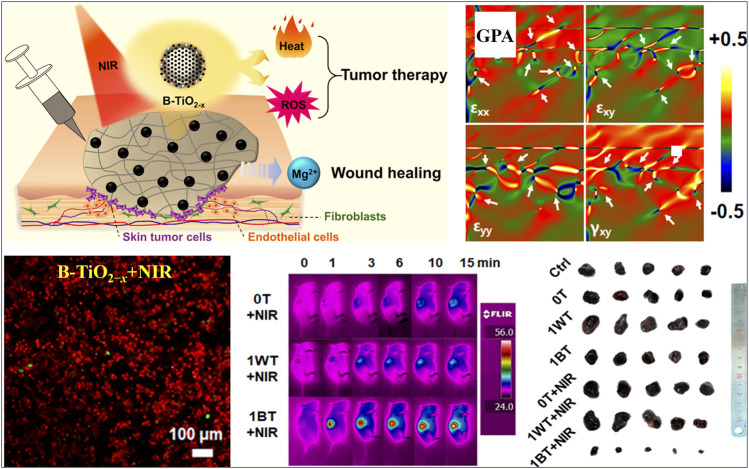
Illustration on action mechanism of B-TiO_2−x_ for synergetic PTT and PDT, geometric-phase analysis (GPA) of B-TiO_2−x_ (white arrows indicate the position of atomic dislocations), calcein AM and PI co-staining of B16F10 melanoma cells after the treatment with B-TiO_2−x_ and NIR irradiation, infrared thermal images of B16F10 tumor-bearing mice after various treatments, and representative photographs of the dissected tumors. Reproduced with permission from Wang X. et al. (2019). Copyright © 2019 American Chemical Society.

Due to the complexity, heterogeneity, and proliferative activity of tumors, the development of multimodal synergistic therapy based on the cooperatively enhanced interactions between PTT and PDT or between phototherapy and other treatments (e.g., chemodynamic therapy), as well as designing more effective multifunctional nanomaterials, are both critical (Fan et al., 2017). The oxygen vacancy engineering provides a new strategy for constructing multifunctional nanomaterial and achieving efficient synergistic therapy. Thus, some oxygen vacancy-engineered nanomaterials have been developed to efficiently combine PDT and PTT to improve the phototherapeutic efficacy against tumor (Sun et al., 2019; Wang X. et al., 2019; Maleki et al., 2021; Yang et al., 2021). For example, Xu et al. (Yang et al., 2021) synthesized the core−shelled TiO_2_@red phosphorus nanorods (TiO_2_@RP NRs) as a nanophotosensitizer for synergetic PDT and PTT, through the vaporization-deposition (VP) method. It can be observed that more oxygen vacancies were created in nanosized TiO_2_ core after the vaporization-deposition, which led to a significantly enhanced NIR absorption in the range of 640–1,100 nm. This enabled TiO_2_@RP NRs to effectively kill significant numbers of deep-tissue tumor cells by generating local heat and ROS, and cause low damage to normal cells. Another example, Wu et al. (Wang X. et al., 2019) reported that Mg-containing black titania (B-TiO_2−*x*_) nanoparticles were synthesized via the Mg-thermic reduction of TiO_2_, exhibiting a crystalline/amorphous core−shell structure with abundant oxygen vacancies within the amorphous shell (Figure 4). The existence of abundant oxygen vacancies in the disordered surface layers (Figure 4) caused the band gap narrowing, giving rise to that the light absorption of B-TiO_2−*x*_ could be effectively extended from the UV to NIR regions. This endowed the B-TiO_2−*x*_ with simultaneous PTT and PDT effects under single-wavelength NIR laser irradiation, resulting in an outstanding curative effect on skin tumors *in vitro* and *in vivo* (Figure 4). Meanwhile, the Mg^2+^ ions released from B-TiO_2−*x*_ could stimulate the proliferation, adhesion and migration of normal skin cells, so as to promote wound healing process.

In recent years, chemodynamic therapy (CDT) represents an especially promising anticancer strategy, and utilizes transition metal oxide catalysis or the transition metal oxide-catalyzed Fenton reaction to promote the transformation of tumor-specific H_2_O_2_ into ROS, thus inducing tumor cells apoptosis (Liu C. et al., 2019; Tang et al., 2019; Ji S. et al., 2021; Tang et al., 2021). Although CDT is effective in preventing tumor growth and metastasis, it doesn’t have the ability to completely eliminate the tumor because of insufficient endogenous H_2_O_2_ (Liu Y. et al., 2020; Ji S. et al., 2021). Thus, oxygen vacancy-promoted synergistic PTT/CDT or PTT/PDT/CDT have been explored to achieve the satisfactory anticancer efficacy, so as to effectively eliminate tumor and inhibit tumor recurrence (Ji et al., 2019; Peng et al., 2020; Sun et al., 2020; Ji X. et al., 2021; Kong et al., 2021; Zhang et al., 2021; Wang et al., 2022; Zhou et al., 2022). For instance, Tan et al. (Zhou et al., 2022) reported the dark-blue co-intercalated MoO_3−*x*_ (denoted as NH−MoO_3−*x*_) nanobelts via the ball-milling process and then aqueous Na^+^/H_2_O co-intercalation as efficient nanozymes for photothermal-enhanced CDT. A combination study verified that the co-intercalation of Na^+^ and H_2_O into layered MoO_3_ nanobelts can induce the formation of the abundant oxygen vacancies. This can provide more active sites for the adsorption and activation of H_2_O_2_ to produce the more •OH and O_2_, thus improving the catalytic performance of nanozymes. Subsequently, the adsorbed O_2_ was further reduced to •O_2_^−^ through the electron transfer between Mo^5+^ and Mo^6+^ contained in oxygen defective NH−MoO_3−*x*_ nanobelts. Moreover, the abundant oxygen vacancies in NH−MoO_3−*x*_ nanobelts led to the generation of defect energy levels and the narrowing of band gap, boosting its absorption in the NIR-Ⅱ region and thus enabling its good photothermal effect. Notably, the catalytic activity of nanozyme can be further enhanced by NIR-Ⅱ light driven photothermal effect. NH−MoO_3−*x*_@BSA nanobelts were obtained after surface modification of bovine serum albumin (BSA). In addition, oxygen defective NH−MoO_3−*x*_@BSA nanobelts exhibits remarkable synergistic therapeutic efficacy, both *in vitro* and *in vivo*, through tumor microenvironment stimulated generation of multiple ROS and NIR-II photothermal activity. The anticancer efficacy can be further strengthened by triple synergistic therapy built on the cooperative enhancement interactions among three treatments and through oxygen vacancy-engineered multifunctional nanomaterials (Ji et al., 2019; Sun et al., 2020; Ji X. et al., 2021; Kong et al., 2021; Zhang et al., 2021). For example, Ji et al. (Kong et al., 2021) designed and fabricated surface-oxidized arsenene nanosheets (As/As_*x*_O_*y*_ NSs) with type II heterojunction and abundant oxygen vacancies through coupling ball-grinding with probe sonication-based liquid exfoliating processes, showing significant synergistic and photo-enhanced PTT/PDT/CDT. A combination study verified that the As_*x*_O_*y*_ in As/As_*x*_O_*y*_ NSs possessed abundant oxygen vacancies and active As^3+^ and As^5+^ sites caused by oxygen vacancies. The existence of abundant oxygen vacancies in such heterojunction As/As_*x*_O_*y*_ NSs caused the narrowing of band gap, promoted the separation of electron holes, and enhanced the adsorption and activation of oxygen. This gave rise to vast •O^2−^ and ^1^O_2_ production in the PDT process (under 660 nm laser irradiation) and obvious NIR-induced photothermal effect in the PTT process (under 808 nm laser irradiation). Moreover, the portion of As_*x*_O_*y*_ with abundant oxygen vacancies not only can catalyze a Fenton-like reaction of H_2_O_2_ to generate •OH and O_2_, but also inactivate some main anti-oxidants and anti-oxidases to inhibit consumption of ROS. This can directly promote ROS burst and essentially enhance the PDT effect. After polydopamine (PDA) and cancer cell membrane (M) coating, As/As_*x*_O_*y*_@PDA@M NSs were obtained with remarkable biocompatibility and homologous targeting ability. Both *in vitro* and *in vivo* evaluations demonstrated that such oxygen vacancy-promoted As/As_*x*_O_*y*_@PDA@M NSs with type II heterojunction can efficiently cause a ROS production in cancer cells and successfully integrate PTT/PDT/CDT, thus establishing triple synergistic therapy with high anticancer efficacy.

**FIGURE 5:**
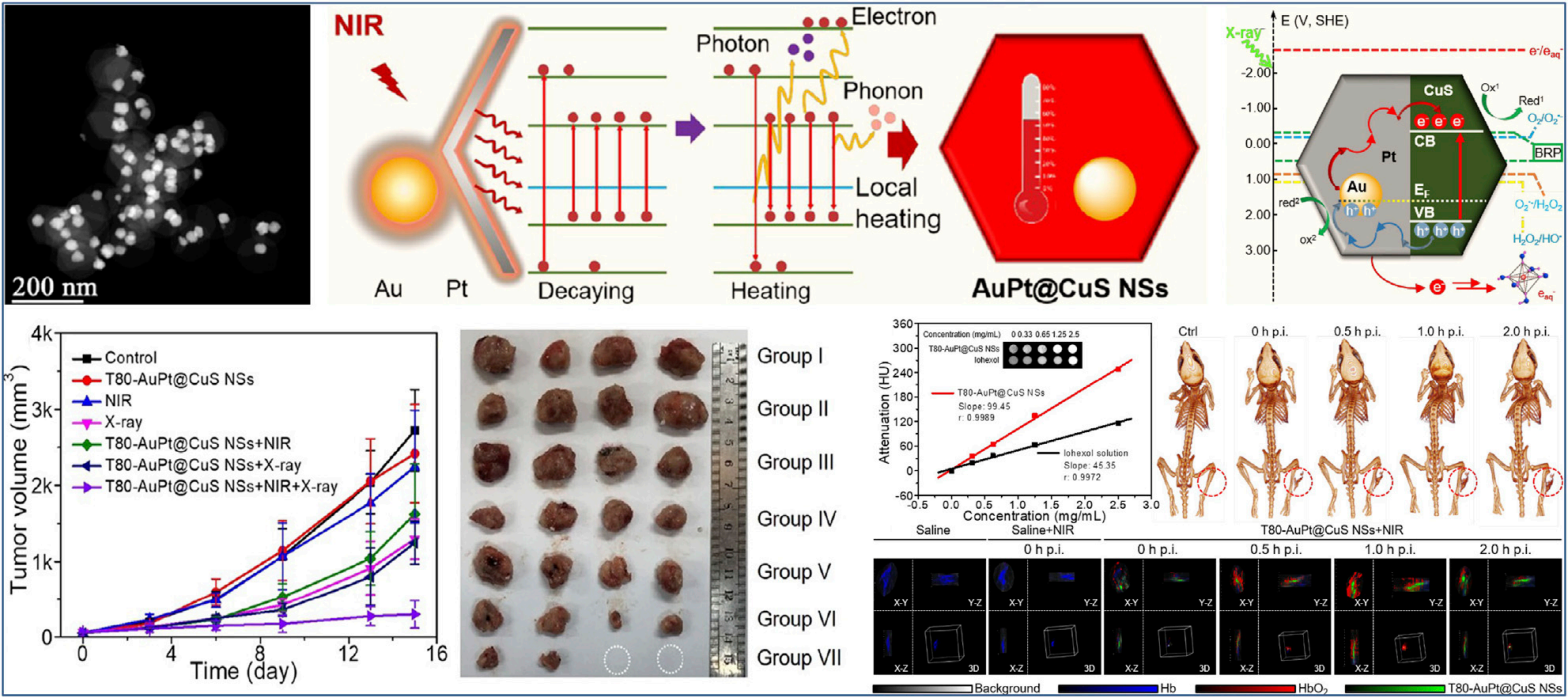
STEM image of AuPt@CuS NSs, schematic illustration of enhanced photothermal mechanism of AuPt@CuS NSs, proposed mechanism of the enhanced deposition of radiation energy by AuPt@CuS NSs, tumor growth curves of 4T1 tumor-bearing mice after various treatments, representative photographs of the dissected tumors, and evaluation of dual modal CT/PA bioimaging capability of T80-AuPt@CuS NSs. Reproduced with permission from Cai et al. (2021). Copyright © 2021 American Chemical Society.

In addition to metal oxide nanosemiconductors, metal sulfide nanomaterials, such as MoS_2_, FeS_2_, and Bi_2_S_3_, have attracted extensive attention in the field of phototherapy, because of their superb electronic and optical properties, good biocompatibility, and easily functionalized surface structures (Meng et al., 2016; Cheng et al., 2018; Zhou et al., 2020; Liu et al., 2021). It is worth noting that sulfur vacancy, as another typical anion vacancy, has a profound impact on the microstructures, physicochemical properties and corresponding phototherapeutic efficacy of metal sulfide nanomaterials (Meng et al., 2016; Lu L. et al., 2018; Cheng et al., 2018; Guan et al., 2018; Ding et al., 2019; Hou et al., 2020; Bai et al., 2021; Cai et al., 2021; Wen et al., 2021). Tan et al. (Cai et al., 2021) reported that a novel plasmonic AuPt@CuS heterostructure toward synergistic PTT and radiotherapy (RT) were formed by decorating AuPt nanoparticles onto the surfaces of CuS nanosheets (Figure 5). It was found that Au and Pt atoms can strongly bond to S atoms, leading to the formation of sulfur vacancy at the surface of CuS NSs. These interfacial sulfur vacancies enabled an increase of carrier density and then induce intensive surface plasmon resonance effect and strong resonance absorption of NIR light (Figure 5). This resulted in the significantly enhanced photothermal performance of AuPt@CuS NSs upon NIR irradiation. Moreover, the presence of sulfur vacancies in AuPt@CuS NSs promoted radiogenerated electron−hole separation, thus resulting in intracellular glutathione (GSH) depletion and ROS generation. This can give rise to enhanced local radiation energy deposition (Figure 5). T80-AuPt@CuS NSs were obtained after surface modification of Tween 80. Importantly, the T80-AuPt@CuS NSs displayed synergistic therapeutic efficacy *in vitro* and *in vivo* upon the combination of PTT and RT, completely killing tumor tissues without later recurrence (Figure 5). In addition, T80-AuPt@CuS NSs possessed the excellent capability of dual-modal computerized tomography (CT)/photoacoustic (PA) imaging, which can effectively guide synergistic radiophotothermal therapy (Figure 5). Another example, Leong et al. (Ding et al., 2019) demonstrated a facile bottom-up route to synthesize sulfur vacancy-defective MoS_2_ quantum dots (QDs, the uniform sizes of around 3.9 nm) with mild aqueous and room temperature conditions. It is worth noting that the degree of sulfur vacancies can be controlled via tuning precursor stoichiometry, thus providing a window of opportunity to adjust their optical and electrical properties. A strong positive correlation between the degrees of sulfur vacancies and the ^1^O_2_ quantum yields (photochemical related ^1^O_2_ generation capacity) can be observed in the obtained MoS_2_ QDs. Further an experimental-computational combination study revealed that the sulfur vacancies in MoS_2_ QDs reduced band gap and strengthened binding affinity between MoS_2_ and ^3^O_2_. This may have contributed to the intersystem crossing and energy transfer separately in the photosensitizing process, thus leading to the observed enhanced ^1^O_2_ generation. Importantly, the resulting MoS_2_ QDs utilizing tunable defect engineering showed more photodynamic efficiency for killing cancer cells, thereby enhancing their therapy effect while preventing the potential chronic side effects.

### Cation vacancy-promoted nanomaterials for phototherapy and multimodal synergistic phototherapy

Metal cation vacancy widely existing in transition metal nanomaterials (e.g., metal chalcogenides, metal oxides, and metal carbides) greatly affects their optical, optothermal, semiconductor, and plasmonic properties, and therefore largely dominates their efficacy of phototherapy (Coughlan et al., 2017; Wang Y. et al., 2019; Wang J. et al., 2020; Zhou B. et al., 2021). Typically, the copper chalcogenide (Cu_2*−x*_X, X = S, Se, Te, 0 ≤ *x* ≤ 1) nanomaterials can support local surface plasmon resonance (LSPR) from NIR to middle infrared region and the characteristic features of *p*-type semiconductor, due to Cu vacancies in these nanomaterials, rendering them promising nanomaterials for phototherapy applications (Li et al., 2015; Ji et al., 2016; Zhang et al., 2016; Lu Y. et al., 2018; Hu et al., 2019; Lai et al., 2019; Wu et al., 2021; Zheng et al., 2021). The free carrier concentration of Cu_2−*x*_S increases with increasing number of Cu vacancies (*x*), which gives rise to a broad and strong absorption band in the NIR region and an enhanced photothermal effect (Coughlan et al., 2017; Huang et al., 2017; Zhao et al., 2020). For example, Ma et al. (Zheng et al., 2021) fabricated the ultrathin starch-coated CuS nanocrystals (CuS NCs) with precisely tunable size and LSPR through a simple and rapid microwave-assisted synthesis strategy, exhibiting a prominent photothermal therapeutic effect on human prostate tumor. The plasmonic analysis and simulation revealed that the obtained CuS NCs possessed an increase in the free carrier density while a decrease in the mean free path of carriers, due to high Cu vacancy concentration and starch coating on CuS NCs. This led to a high LSPR absorption of CuS NCs at the NIR wavelength 980 nm and markedly enhanced their photothermal performance. Furthermore, it was found that the free carrier densities of small CuS NCs were higher than that of large CuS NCs because of their structural differences, so small CuS NCs had the higher photothermal performance at 980 nm. In addition, small CuS NCs as efficient photothermal agents displayed a high photothermal ablation efficacy for human prostate cancer PC-3/Luc+ cells and an enhanced biocompatibility. Another example, Wei et al. (Wu et al., 2021) constructed an ultrathin Cu-loaded CoCuFe-selenide (CCFS) through *in situ* selenylation treatment of CoCuFe-layered double hydroxide (CoCuFe-LDH) precursor, which showed vastly enhanced photothermal conversion efficiency for NIR-triggered PTT. It was found that more Cu vacancies and more free charge carriers can be formed in these selenides via regulating the Cu doping ratio or/and using surface corrosion. Specifically, more Cu vacancies on the surface of the CCFS can be generated with an increase of Cu content from 10% to 30%, while Cu vacancies gradually increased as the pH decreased from 7.4 to 5.4. Correspondingly, CCFS with a 30% Cu doped ratio at pH 5.4 showed maximum Cu vacancies concentration and strongest LSPR absorbance characteristic in the NIR-Ⅰ region, thus leading to an outstanding photothermal conversion efficiency of 81.0% at 808 nm laser irradiation. Moreover, the surface of CCFS can be further modified with polyvinyl pyrrolidone (PVP) and L-arginine (L-Arg) to obtain CCFS-PVP-L-Arg (CPA) system, permitting localized NO gas therapy (GT) in the tumor site through L-Arg decomposition activated by endogenous H_2_O_2_. In addition, both *in vitro* and *in vivo* studies have confirmed that the CPA nanocomposites showed excellent synergetic PTT and GT for tumors under NIR irradiation. LSPR coupling effect between noble metal and cationic vacancy defective Cu_2−*x*_S can significantly enhance NIR-II-driven PTT. Zhang et al. (Ji et al., 2016) constructed an Au@Cu_2−*x*_S core–shell nanorods (Au@Cu_2−*x*_S NRs) by the non-epitaxial strategy of tributylphosphine-initialized cation exchange, showing strong LSPR coupling effect and enhanced photothermal conversion efficiency under 1,064 nm light irradiation. It was found that LSPR of Au core in Au@Cu_2−*x*_S NRs originated from the collective oscillations of electrons. In contrast, the collective oscillations of holes led to LSPR of Cu_2−*x*_S shell, due to abundant Cu vacancy induced *p*-type carriers. Based on experimental and theoretical studies, the formation of Au@Cu_2−*x*_S core–shell nanorods could couple the LSPR properties of Au and Cu_2−*x*_S NRs to a maximum degree, resulting in a plasmon-enhanced absorption in NIR-II region and heightened photothermal conversion efficiency. Thus, Au@Cu_2−*x*_S NRs showed a very strong capacity on killing HeLa cell under l064 nm laser irradiation.

Due to the presence of Cu vacancies in the lattice, abundant free carriers, excess holes, and more Cu^+^ active sites can be created in the Cu_2−*x*_S semiconductor nanocrystals, which contributes to the generation of ROS (Coughlan et al., 2017; Zhao et al., 2020). Thus, these Cu_2−*x*_S can also be designed as the multifunctional nanomaterials for synergistic tumor therapy (e.g., PTT/CDT, PTT/PDT, and PTT/PDT/CDT) (Lu Y. et al., 2018; Hu et al., 2019; Lai et al., 2019). Wu et al. (Hu et al., 2019) reported that the ultrasmall PEG-modified Cu_2−*x*_S nanodots (Cu_2−*x*_S-PEG NDs) with abundant Cu vacancies were fabricated for photoacoustic imaging-guided synergistic therapy against tumors, through a redox reaction between sulfur and Cu(acac)_2_ and subsequent DSPE-PEG2000-NH_2_ surface coupling. It was observed that as-obtained ultrasmall Cu_2−*x*_S-PEG NDs (their average particle size ≤5 nm) can be efficiently and passively accumulated at the tumor sites through the typical EPR effect. Especially, these Cu_2−*x*_S-PEG NDs at the tumor sites can effectively catalyze tumor-overexpressed H_2_O_2_ to produce a large amount of cytotoxic •OH radicals, thus inducing tumor cell apoptosis. The findings were likely to be attributed to the formation of abundant Cu^+^ active sites in Cu_2−*x*_S-PEG NDs induced by Cu vacancies, improving the catalytic activity of Cu_2−*x*_S-PEG NDs for Fenton reaction. Moreover, the presence of Cu vacancies in Cu_2−*x*_S-PEG NDs caused also their LSPR and strong light absorption in NIR-II biowindows. These Cu_2−*x*_S-PEG NDs, thus, showed significantly enhanced photoacoustic imaging and photothermal effect, which could efficiently monitor the tumor regions and promote photothermal ablation for tumors as well as synergistically enhance Fenton-mediated therapeutic efficacy. In addition, both *in vitro* and *in vivo* experiments further demonstrated that Cu_2−*x*_S-PEG NDs had good biocompatibility and outstanding synergetic therapeutic efficacy, providing a new strategy of photoacoustic imaging-guided synergistic therapy for potential clinical application. Another example, Zhang et al. (Lai et al., 2019) synthesized the peptide nanotube-supported Cu_2−*x*_S nanoparticles (CuS−PNTs) with uniform particle size and ordered dispersion. The as-synthesized CuS−PNTs had obvious absorption in the NIR region and significantly enhanced photothermal effect, due to the strong LSPR originated from Cu vacancies in the lattice. Notably, taking the advantage of type I PDT mechanism, CuS−PNTs with the characteristics of *p*-type semiconductor can induce the efficient generation of •O^2−^ under NIR laser irradiation, and then the •O^2−^ can be converted into cytotoxic •OH through disproportionation reaction and subsequent Cu^+^-catalyzed Fenton-like reaction. This resulted in a synergetic PTT and PDT for tumors treatment. Moreover, CuS−PNTs can further be covalently grafted with an oxaliplatin prodrug (Pt−CuS−PNTs) to construct a multifunctional nanoplatform for combined photo- and chemotherapy. Correspondingly, the antitumor studies have confirmed that Pt−CuS−PNTs can significantly inhibit the tumor growth and lung metastasis of melanoma.

**FIGURE 6:**
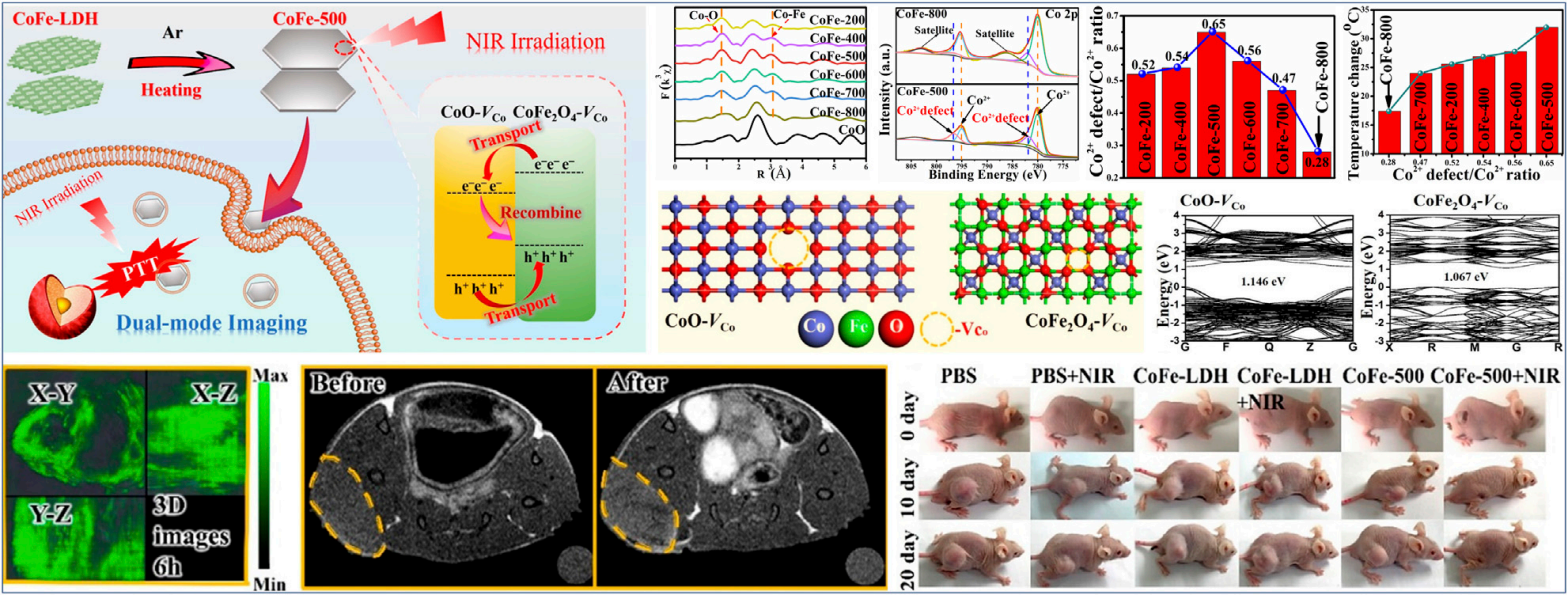
Schematic Illustration of the synthesis of CoFe-500 for PA/MR/NIR imaging-guided PTT, Fourier-transform EXAFS spectra at Co K-edge for various CoFe-x samples, Co 2p XPS spectra for CoFe-500 and CoFe-800, Co^2+^ defect/Co^2+^ peak area ratio for various CoFe-x samples, and optimized geometries for a CoFe-500 bulk heterojunction composed of CoO-V_Co_ and CoFe_2_O_4_-V_Co_, band structures of CoO-V_Co_ and CoFe_2_O_4_-V_Co_, 3D PAI images of tumor site at 6 h postinjection, *in vivo* T1-MRI imaging of HeLa-bearing mice before and after injected with CoFe-500, and photographs at different times after various treatments. Reproduced with permission from Wang L. et al. (2021). Copyright © 2020 American Chemical Society.

For other transition metal nanomaterials such as metal oxides, metal hydroxides, and metal carbides, metal cation vacancy also plays a key role in tailoring microstructure and improving therapeutic efficacy (Naldoni et al., 2012; Li et al., 2018; Wang J. et al., 2020; Zhou B. et al., 2021; Li et al., 2022). Guan et al. (Wang J. et al., 2020) reported that a series of vacancy defects-promoted CoFe-mixed metal oxides (CoFe-*x*, *x* represented different calcination temperatures) as efficient photothermal agents were successfully fabricated on the basis of the precise control over the calcination process of CoFe-LDH precursors (Figure 6). A combination study including HRTEM, EXAFS, and XPS verified that the morphology (e.g., particle size) and microstructure (e.g., the concentration of Co^2+^ vacancies) of CoFe-*x* nanoagents can be effectively adjusted through tuning different calcination temperature of CoFe-LDH precursors (Figure 6). In particular, with the increase of calcination temperature from 200 to 800°C, the concentration of Co^2+^ vacancies increased first and then decreased, and the maximum Co^2+^ vacancies concentration was present in the sample of CoFe-500 (Figure 6). The optimized nanoagent (CoFe-500, with the highest concentration of Co^2+^ vacancies) via tuning the calcination temperature of CoFe-LDH precursors and Co^2+^ vacancies concentration gave a most efficient photothermal performance under NIR irradiation (Figure 6). Experiments and DFT calculations revealed that Co^2+^ vacancies greatly affected the electronic structure of CoFe-*x* and resulted in the narrowing of the band gap (Figure 6), so as to increase the non-radiative recombination rate and enhance the NIR-driven photothermal effects. Moreover, *in vitro* and *in vivo* results indicated that CoFe-500 had outstanding photothermal therapeautic efficacy, while also acting as a promising agent for biomedical imaging such as near-infrared thermal imaging, magnetic resonance, and photoacoustic imaging (Figure 6). Another example, Chen et al. (Zhou B. et al., 2021) reported that a distinct BSA-modified W_1.33_C nanosheet (W_1.33_C-BSA) with ordered divacancies was efficiently fabricated through designing a parent bulk laminate in-plane ordered (W_2/3_Y_1/3_)_2_AlC ceramic and optionally etching Al and Y elements. Especially, both theoretical simulations and experiments revealed that W_1.33_C-BSA nanosheets with ordered divacancies had a broad and strong absorption band in the NIR region, and resulted in excellent photothermal conversion efficiency under both the NIR-I and NIR-II laser irradiation. Notably, W_1.33_C-BSA nanosheets could be rapidly degraded in normal tissue and easily excluded from the body due to abundant ordered divacancies in these ultrathin nanosheets, while their unique pH-responsive characteristics enabled them to enrich and remain longer at tumor sites. Moreover, benefiting from the superior X-ray attenuation ability and high NIR absorbance of these nanosheets, W_1.33_C-BSA nanosheets exhibited highly effective dual-modal PA/CT imaging-guided photothermal therapy against tumors.

### Carbon vacancy-promoted nanomaterials for phototherapy and multimodal synergistic phototherapy

In addition to transition metal nanocompounds, carbon-based nanomaterials, including but not limited to graphene, carbon quantum dots, and derivatives of metal−organic frameworks (MOFs), are also promising candidates as nanomaterials for phototherapy (Lu L. et al., 2018; Chen et al., 2021; Tamtaji et al., 2021; Tian et al., 2021). Meanwhile, carbon vacancy defects in carbon-based nanomaterials can significantly influence their configuration and atomic arrangements and effectively regulate the local π-electron system, so as to tailor the global properties of phototherapeutic nanomaterials and to achieve desired phototherapy effects (Liang et al., 2015; Li et al., 2017; Liu H. et al., 2020; Weng et al., 2020; Wang L. et al., 2021; Chang et al., 2021; Chen et al., 2021; Guo et al., 2021; Tamtaji et al., 2021). Guan et al. (Weng et al., 2020) reported that a series of vacancy defective nanocarbon polyhedral-supported Cu nanoparticles (Cu@CPP-*t*, *t* represented different calcination temperatures) as novel photothermal agents were successfully prepared through the controlled calcination of Cu-based MOF (Cu-BTC) precursors at temperatures from 400 to 900°C Specifically, Cu@CPP-*t* samples comprised partially oxidized Cu nanoparticles dispersed on nanocarbon polyhedra, and their nanocarbon supports were semigraphitic phase with abundant carbon vacancies. It was worth noting that the concentration of carbon vacancies increased with calcination temperature, arising from gradually enhanced interaction between Cu nanoparticles and nanocarbon polyhedral, while the size of the Cu nanoparticles also increased with increasing calcination temperature. At the same time, it can be found that the photothermal effects of as-obtained Cu@CPP-*t* samples were strongly and positively correlated with the concentration of carbon vacancies and the size of copper nanoparticles in the calcination temperature range of 400–800°C. In particular, Cu@CPP-800 sample showed significantly enhanced photothermal conversion efficiency under NIR-Ⅰ laser irradiation. The above results were probably due to the following reasons: 1) more carbon vacancies boosted π→π* transitions in the semigraphitic nanocarbon polyhedral, inducing stronger absorption of incident photons; 2) more carbon vacancies as recombination centers promoted non-radiative relaxation processes and thus increased heat release; 3) while Cu LSPR absorptions also enhanced with the increasing of Cu nanoparticle size. However, on increasing the calcination temperature from 800 to 900°C, the photothermal performance decreased due to excessive sintering of the Cu@CPP-900 samples. Thus, increasing the concentration of carbon vacancies together with Cu nanoparticle size via tuning calcination temperature was an effective way to enhance photothermal performance. Moreover, *in vitro* and *in vivo* studies confirmed that Cu@CPP-800 was a very effective agent for photothermal cancer treatment and also NIR/photoacoustic bioimaging. Another example, Wang et al. (Liu H. et al., 2020) reported that a type of graphene quantum dots (9T-GQDs) with strong NIR-II absorption was synthesized by a one-step solvothermal treatment using phenol and hydrogen peroxide as precursor under a high magnetic field with an intensity of 9T. It can be observed that the dissolved oxygen from H_2_O_2_ decomposition under external high magnetic field induced the crystallization of small carbon fragments, leading to the formation of abundant vacancy defects in the GQDs. Notably, with the increase in magnetic field intensity, dissolved oxygen and resulted vacancy defects increased. Carbon atoms located round vacancy defects showed high coordination unsaturation and high reactivity, so the defective carbon atoms were easy to react with the superoxide anion and hydroxyl radical intermediates, forming abundant C=O quinone-type groups and C−OH phenol groups on the surface of GQDs. On the one hand, abundant C=O bonds on the 9T-GQDs led to the formation of larger conjugated system containing plentiful delocalized π electrons, thus giving rise to the strong NIR-II absorbance of 9T-GQDs and high photothermal conversion efficacy. On the other hand, abundant C−OH groups as hydrophilic groups ensured small size distribution (3.6 nm), good water-solubility and excellent biocompatibility of 9T-GQDs. Both *in vitro* and *in vivo* studies confirmed that as-obtained 9T-GQDs could effectively ablate tumor cells and inhibit the tumor growth under NIR-II irradiation. In addition, the 9T-GQDs displayed enhanced NIR-II imaging of tumor, suggesting a great potential of 9T-GQDs for NIR-II imaging-guided PTT.

## Conclusion and prospects

Phototherapy including PDT and PTT is a non-invasive light-induced therapy, which uses NIR-responsive nanomaterials to achieve effective local treatment in tumor sites and could diminish damage to normal tissues. Moreover, multimodal synergistic phototherapy (including synergistic PTT and PDT as well as combined phototherapy and other therapies) based on multifunctional nanomaterials can achieve synergistic enhancement of tumor treatment. It is worth noting that the phototherapy is a nanomaterial microstructure (microscopic electronic and geometric structures) sensitive process, and the effectiveness of phototherapy depends largely on nanomaterials. The vacancy-type defects, such as oxygen vacancy, metal vacancy, carbon vacancy, and sulfur vacancy, commonly exist in phototherapeutic nanomaterials, which can greatly influence and directly change the microstructure, properties and phototherapeutic efficacy of nanomaterials. At the same time, the rational design and preparation of vacancy defective nanomaterials as well as the roles of vacancy defect in phototherapy have been paid more and more attention. Therefore, this review focuses on the recent vacancy defect chemistry research and vacancy engineering strategy, as well as efficient vacancy defective nanomaterials and their role in phototherapy and multimodal synergistic phototherapy. The vacancy defect chemistry has become a significant research direction to design and prepare efficient nanomaterials for phototherapy. The existence of vacancy defects in phototherapeutic nanomaterials will disturb the chemical state of the surrounding atoms or ions to some extent and induce lattice distortion/strain. This, thus, can effectively change the local bond, atomic coordination and electronic properties around specific atomic positions located at vacancy, bringing some unpredictable properties (e.g., outstanding optical, optothermal, optoelectronic, semiconductor, and plasmonic properties). Specifically, constructing vacancy defects in phototherapeutic nanomaterials can induce defect energy levels and thus effectively regulate their electronic structures and band gap states, give rise to the strong LSPR absorption in the NIR region, and promote the adsorption and activation of oxygen species. These will result in enhanced photoinduced ROS production efficiency (ROS quantum yield) and high photothermal conversion of phototherapeutic nanomaterials. Moreover, with the deepening of the research on vacancy defect chemistry, vacancy defect engineering for synthesizing various phototherapeutic nanomaterials with significantly improved and optimized performance has been developed. Notably, vacancy engineering not only provide a comprehensive insight into the understanding and utilization of vacancy defect chemistry in phototherapeutic nanomaterials, but also offer a novel and effective technique to regulate/optimize the microstructure, characteristics and phototherapeutic efficacy of nanomaterials. The construction methods of vacancy defects in phototherapeutic nanomaterials can generally be divided into two broad categories: 1) directly synthesizing vacancy defective nanomaterials; 2) post treatment of nanomaterials to construct vacancies. In terms of the former method, the specific vacancies can be directly induced with the crystal growth by tuning crystallization conditions and methods, or by regulating the ratio of starting materials, or by elemental doping. While the latter post treatment method like annealing, irradiation, ball-milling, etching, and redox is also an efficient and controllable vacancies construction strategy. Additionally, a deeper and more detailed understanding of the influence and role of vacancy defects on phototherapy and multimodal synergistic phototherapy is also crucial to rational design of new phototherapeutic nanomaterials, effective enhancement of their therapeutic efficacy, and systematic development of phototherapeutic applications.

Although more and more studies on vacancy defect-promoted nanomaterials for phototherapy and multimodal synergistic phototherapy have been reported, there are still many scientific and engineering topics to encourage researchers to further explore. First, although these vacancy defect-promoted nanomaterials have been proved to be high anticancer efficacy via preclinical research, their metabolic behavior, potential toxicity and biological effects still need to be deeply and systematically studied through *in vitro* and *in vivo* experiments. This will further promote optimization of phototherapeutic nanomaterials and improve their therapeutic outcome, providing an advanced platform for future clinical applications. Second, although the above vacancy engineering strategies have been able to construct various vacancies in phototherapeutic nanomaterials, it is still necessary to find “clean chemistry methods”, that is, more simple, efficient, and controllable vacancy defect construction methods. This will allow large-scale preparation of vacancy defect-promoted nanomaterials, in order to meet the large demand for advanced phototherapeutic nanomaterials in future clinical applications. Third, carbon-based nanomaterials with abundant vacancies, such as vacancy engineered carbon dots and graphenes, should be vigorously developed for future clinical translation, due to their well-defined microstructure, multifunctionality, superior stability, good biocompatibility, and easy manufacturing. Finally, in addition to the application in the field of phototherapy and anticancer, vacancy defect-promoted nanomaterials should also be been used in a wider range of biomedical applications, such as biosensing, antibacterials, and antivirals (Macharia et al., 2021; Ren et al., 2022).

In conclusion, vacancy defect chemistry and engineering of phototherapeutic nanomaterials, the role of vacancy defective nanomaterials on phototherapy and their phototherapeutic applications is systematically summarized in this review. In general, the abundant physicochemical properties induced by vacancies, coupled with the recent success in the design of advanced functional nanomaterials for phototherapeutic applications, inspire growing research interest in vacancy engineering and vacancy-engineered nanomaterials for using phototherapy. Therefore, we sincerely hope that this review can provide a profound understanding and inspiration for the design of advanced phototherapeutic nanomaterials, and significantly promote the development of the efficient therapies against tumour.
